# Supplementary material for: Extracellular Regulated Kinase Phosphorylates Mitofusin 1 to Control Mitochondrial Morphology and Apoptosis
Source: Mol Cell. 2015 Apr 16;58(2):244–54. doi: 10.1016/j.molcel.2015.02.021 (PMC4405354; doi:10.1016/j.molcel.2015.02.021)
Supplement: Document S1. Figures S1–S5 and Supplemental Experimental Procedures [file mmc1.pdf]

Molecular Cell, Volume *58*

Supplemental Information

Extracellular Regulated Kinase Phosphorylates Mitofusin 1 to Control Mitochondrial Morphology and Apoptosis

Aswin Pyakurel, Claudia Savoia, Daniel Hess, and Luca Scorrano

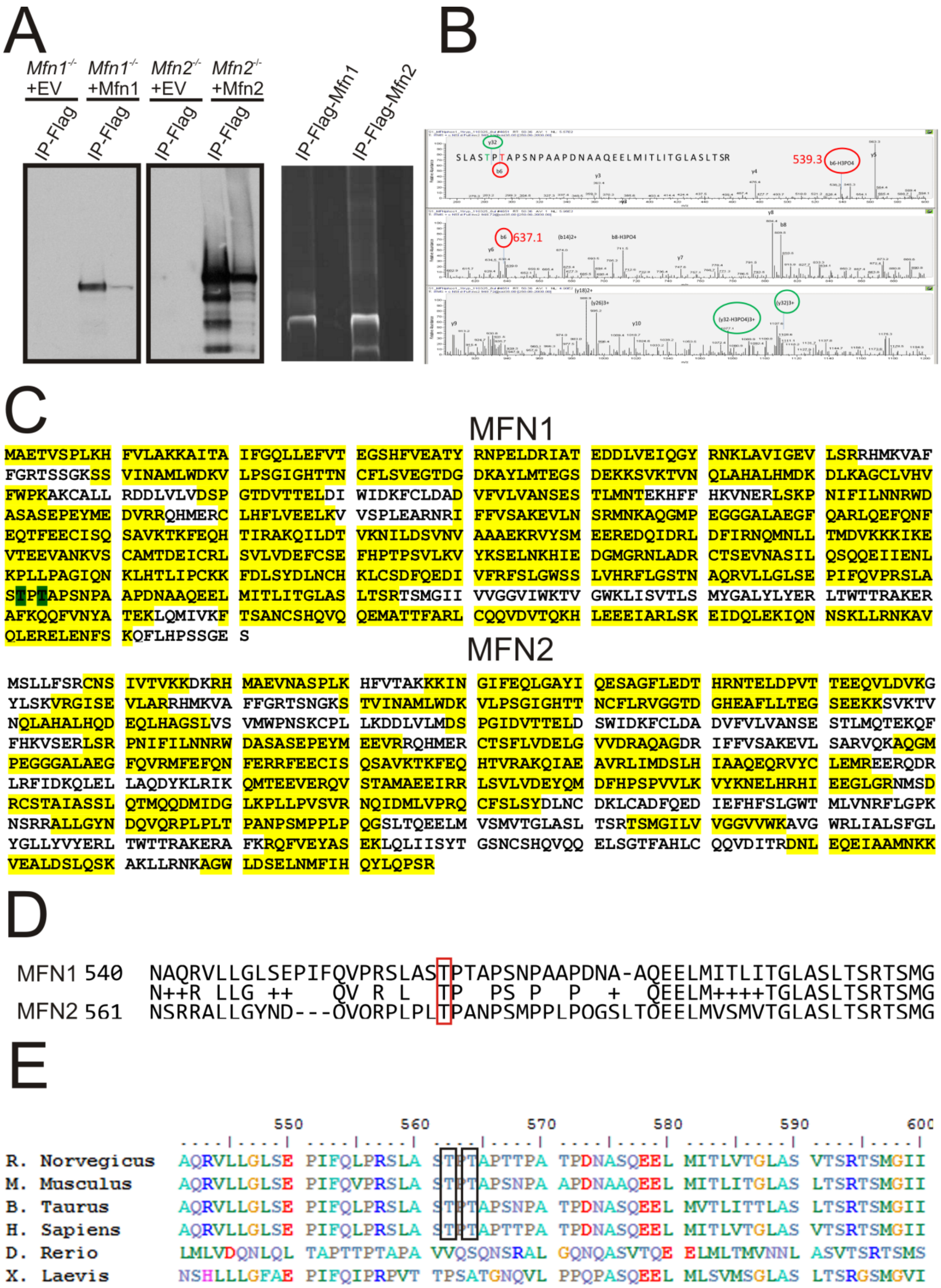

Figure S1. LC-MS/MS Identification of MFN1 Phosphorylation, Related to Fig. 1

(A) FLAG-MFN1 and FLAG-MFN2 were immunoprecipitated where indicated from *Mfn1*$^{-/-}$ and *Mfn2*$^{-/-}$ MEFs transfected as indicated, separated by SDS PAGE and detected by anti-Flag immunoblotting (left panels) or Simply Blue Safe staining (right panel).

(B) MS/MS spectrum of the tryptic peptide of MFN1 containing one phosphorylation either on T562 or T564. Ions highlighted with green circles account for the T562 phosphorylation whereas the ion highlighted with a red circle accounts for the T564 phosphorylation.

(C) LC-MS/MS coverage of the immune-purified FLAG-MFN1 and FLAG-MFN2. Detected peptides are highlighted in yellow, post-translational modifications in green.

(D) ClustalW alignment of the phosphorylated region of MFN1 with MFN2. Box: conserved T

(E) ClustalW alignment of the phosphorylated region of MFN1 of the indicated species. Box: conserved phosphorylated T

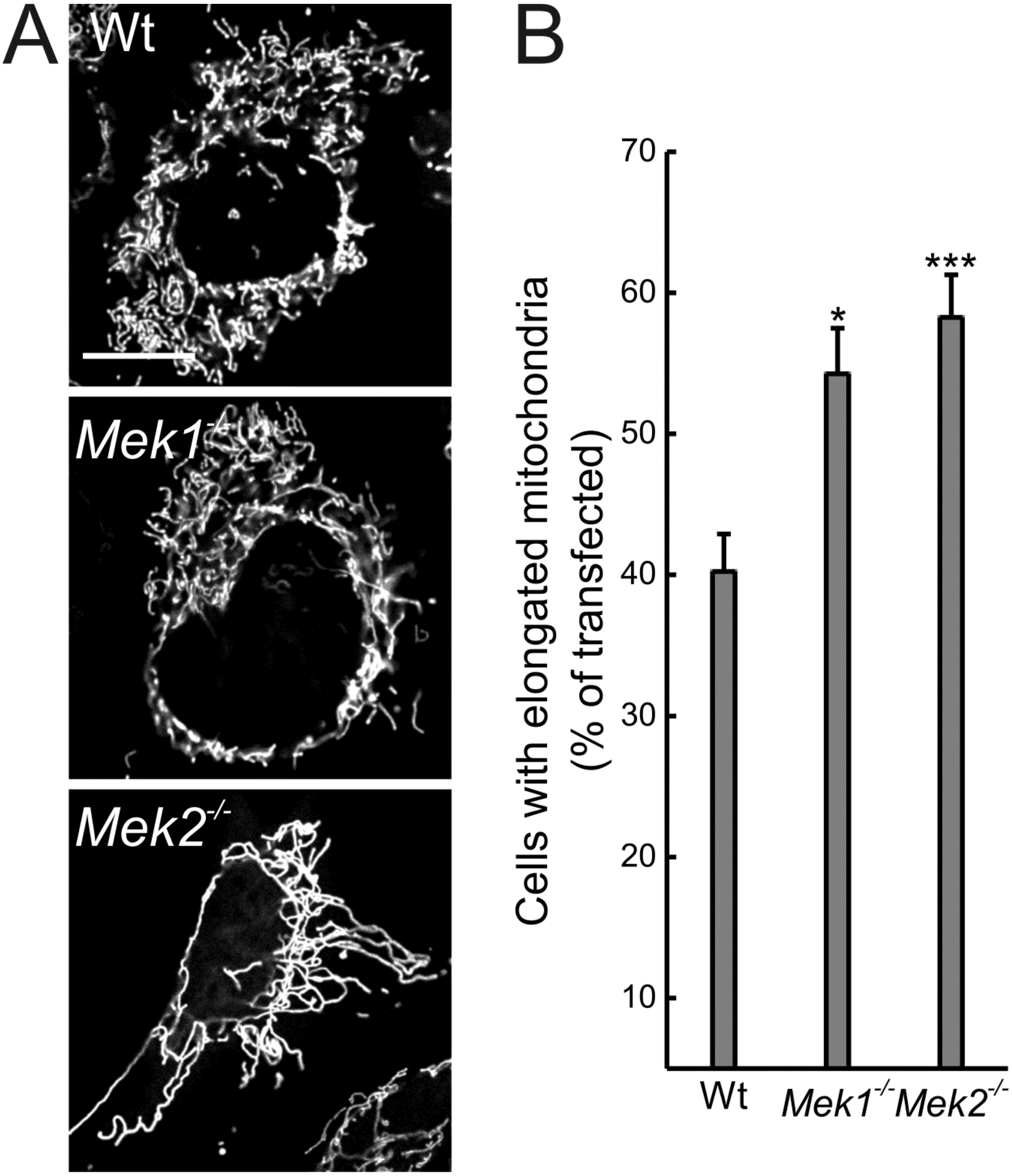

Figure S2. Mitochondria Are Elongated In *Mek1$^{-/-}$* and *Mek2$^{-/-}$* MEFs, Related to Fig. 2

(A) Representative confocal images of mitochondrial morphology in MEFs of the indicated genotype transfected with mtYFP. Scale Bar, 10 µm.

(B) Experiments were carried out as in **A**. Data represent mean±SEM of 5 independent experiments (n=100 cells per condition in each experiment). *P<0.05, **P<0.01, ***P<0.001 in a paired Student's t Test vs. Wt

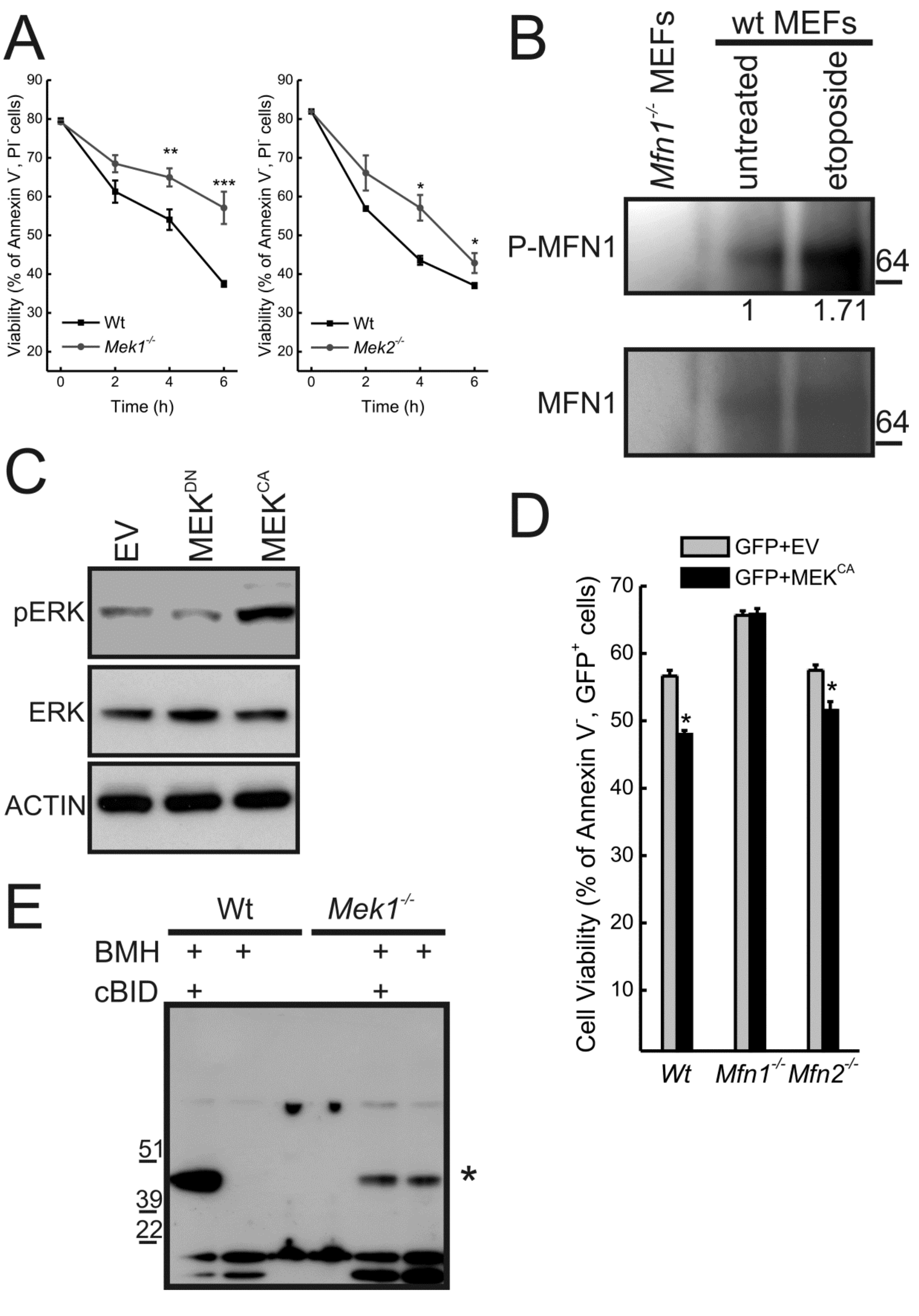

Figure S3. MFN1 Phosphorylation Occurs during and Modulates Cell Death, Related to Fig. 3

(A) MEFs of indicated genotypes were treated with 2 μM staurosporine for the indicated times. Data are mean±SEM of 5 independent experiments. *P<0.05, **P<0.01, ***P<0.001 in a paired Student's t Test vs. Wt

(B) Endogenous MFN1 was immunoprecipitated from MEFs of the indicated genotype treated where indicated with 2 μM etoposide, separated by SDS-PAGE and immunoblotted using the indicated antibodies. The densitometric P-MFN1/MFN1 ratio is indicated, normalized to untreated MEFs.

(C) MEFs were transfected with the indicated plasmids and lysed after 24 hrs. Equal amounts (30 μg) of proteins were separated by SDS-PAGE and immunoblotted using the indicated antibodies.

(D) MEFs of indicated genotypes cotransfected with GFP and the indicated plasmids were treated with 2 μM staurosporine for 6 hours. Data represent mean±SEM of 3 independent experiments. *P<0.05, **P<0.01, ***P<0.001 in a paired Student's t Test vs. Wt

(E) Mitochondria isolated from the indicated genotypes were treated where indicated with cBID and crosslinked with 10mM BMH where indicated. Equal amounts (30 μg) of proteins were analyzed by SDS-PAGE/immunoblotting. Asterisk: BAK multimers.

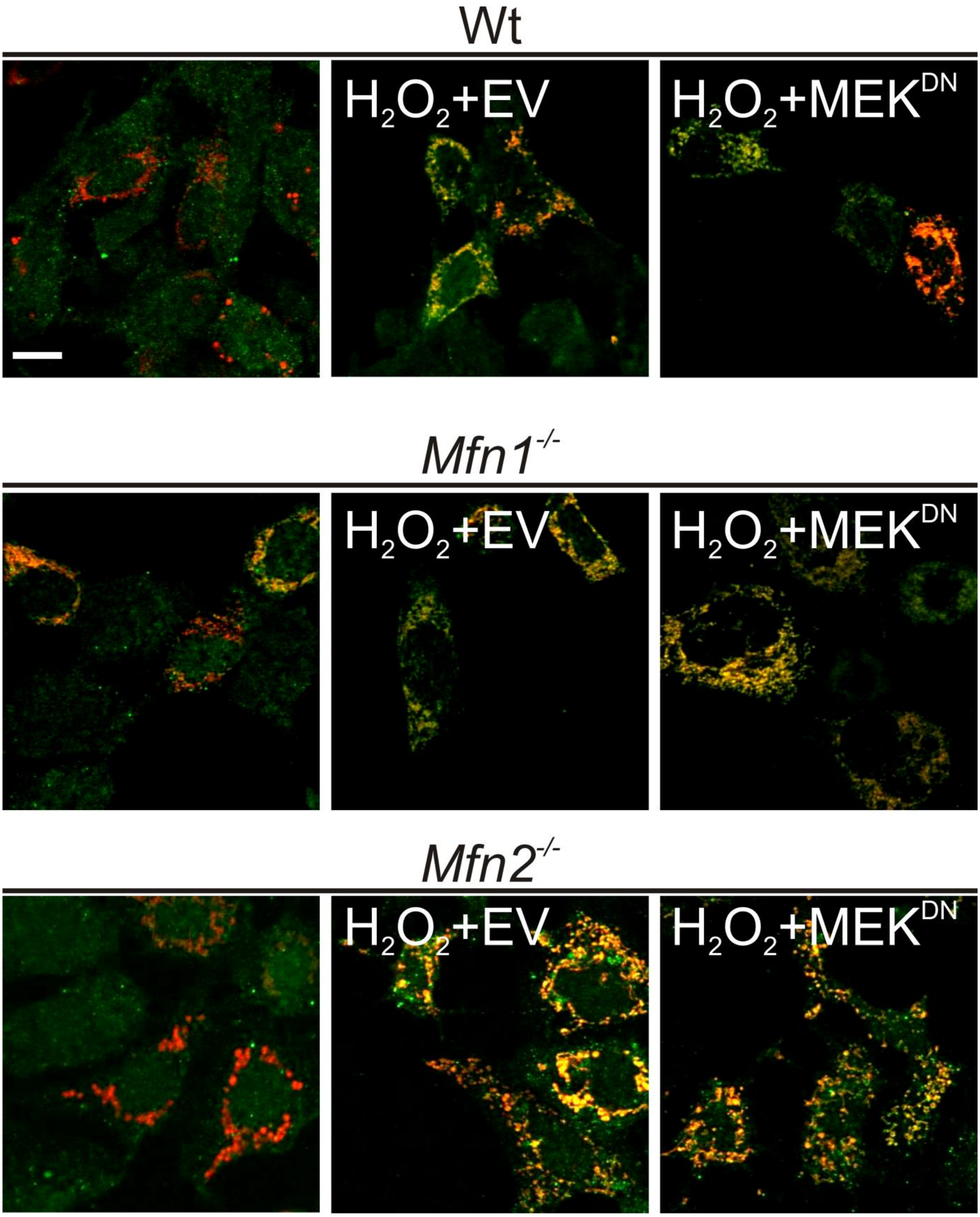

Figure S4. MEK^{DN} Does Not Alter BAX Translocation, Related to Fig. 4.
Representative images of subcellular BAX distribution in MEFs of indicated genotypes cotransfected with mtRFP (red) and the indicated plasmids and treated where indicated for 30 min with 1 mM H_2O_2, fixed and immunostained for BAX (green). Scale Bar, 10 µm.

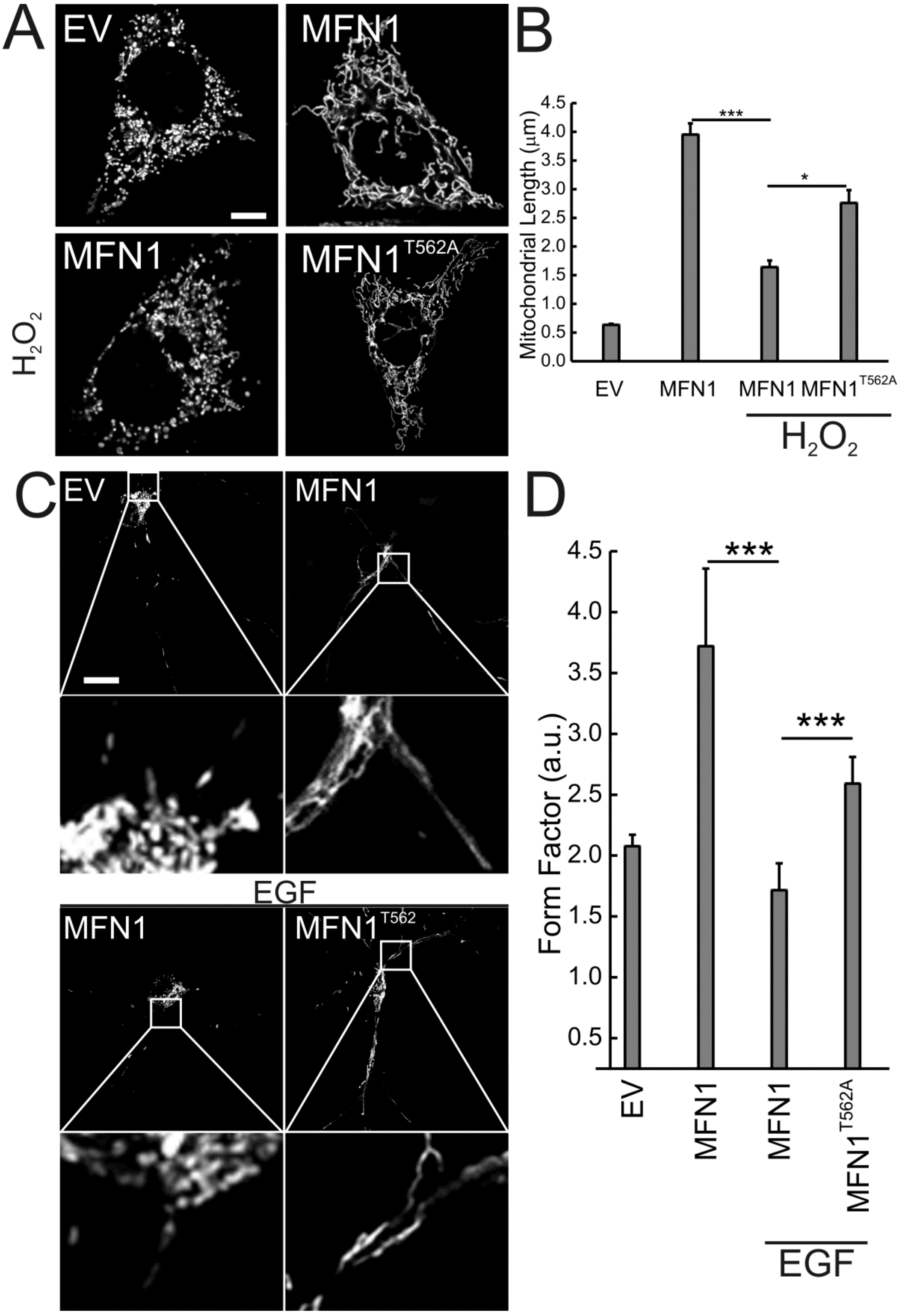

Figure S5. MFN1 Phosphorylation Regulates Mitochondrial Morphology in Oxidative Stress and Primary Cortical Neurons, Related to Fig. 5

(A) Representative confocal images of mitochondrial morphology in MEFs of the indicated genotype cotransfected with mtYFP and the indicated plasmids. Cells were treated with H_2O_2 (250μM for 30 min) where indicated. Scale bar, 10 μm.

(B) Experiments were carried out as in (A). Data represent mean±SEM of 4 independent experiments. *P<0.05, ***P<0.001 in a paired Student's t Test between the indicated treatments

(C) Representative images of primary cortical neurons co-transfected with mtRFP and the indicated plasmids. Where indicated, neurons were treated with 10 nM EGF for 10 min. Scale bar, 5 μm. The boxed areas are magnified 6X.

(D) Quantitative analysis of mitochondrial morphology in experiments carried out as in (A). Data represent mean±SEM of 4 independent experiments. ***P<0.001 in a paired Student's t Test between the indicated treatments.

Supplemental Experimental Procedures

Molecular Biology

For the generation of the Mfn1 mutants, the following primers were used:

T562A F: 5'–GTCCCTAGATCTTTAGCTTCAGCTCCTACTGCTCCTTCTAACC-3'; R: 5'-GGTTAGAAGGAGCAGTAGGAGCTGAAGCTAAAGATCTAGGGAC-3,

T562D F: 5'-CCCTAGATCTTTAGCTTCAGACCCTACTGCTCCTTCTAACC-3'; R: 5'-GGTTAGAAGGAGCAGTAGGGTCTGAAGCTAAAGATCTAGGG-3',

T564A F: 5'-ATCTTTAGCTTCAACTCCTACTGCTCCTTCTAACCCAGC-3'; R: 5'-GCTGGGTTAGAAGGAGCAGTAGGAGTTGAAGCTAAAGAT-3'),

T564D F: 5'-TCTTTAGCTTCAGACCCTGATGCTCCTTCTAACCCAGC-3'; R: 5'-GCTGGGTTAGAAGGAGCATCAGGGTCTGAAGCTAAAGA-3',

T562AT564A F: 5'-GTCCCTAGATCTTTAGCTTCAGCTCCTGCTGCTCCTTCTAACC-3'; R: 5'-GGTTAGAAGGAGCAGCAGGAGCTGAAGCTAAAGATCTAGGGAC-3',

T562DT564D F: 5'-CCCTAGATCTTTAGCTTCAGACCCTGATGCTCCTTCTAACC-3'; R: 5'-GGTTAGAAGGAGCATCAGGGTCTGAAGCTAAAGATCTAGGG-3'.

Liquid chromatography-mass spectrometry

Extracted peptides from gels were injected onto a reversed phase column for liquid chromatography-mass spectrometry (LC-MS) analysis in the information-dependent acquisition mode. Electrospray ionization LC-MS/MS was performed using a Magic C18 HPLC column (75 µm × 10 cm; Swiss BioAnalytics) with a 1200 Nano-HPLC system (Agilent Technologies) connected to a LTQ Orbitrap Velos (Thermo Scientific). The peptides were loaded onto a peptide captrap (Michrom BioResources) at a flow rate of 10 µl / min for 5 minutes. They were eluted at a flow rate of 400 nl / min with a linear gradient of 2 – 36 %

acetonitrile in 0.1 % formic acid (in H_2O) in 30 minutes. Information-dependent acquisition analyses was done according to the manufacturer's recommendations, i.e. 1 survey scan at 60K resolution in the Orbitrap cell was followed by up to 20 product ion scans in the linear ion trap, and precursors were excluded for 15s after their second occurrence. Individual MS/MS spectra, containing sequence information were compared with the program Mascot against the mammalian sub set of the protein sequence database Swiss-Prot 2010_09 (Perkins *et al.*, 1999). Carboxyamidomethylation of cysteine (+57.0245 Da) was set as a fixed modification and phosphorylation of serine, threonine and tyrosine (+79.9663 Da), oxidation of methionine (+15.9949 Da), deamidation of asparagine and glutamine (+0.984016 Da) and Pyro-glu formation on N-terminal glutamine (-17.02655 Da) were set as variable. Parent tolerance was 3 PPM and fragment tolerance 0.8 Da. No enzyme specificity was used for the combined searches of the tryptic and AspN digested samples. The results were further analyzed with Scaffold and ScaffoldPTM (Proteome Software).

Combined oxygen and glucose deprivation (OGD) in primary neurons

Primary cortical neurons were exposed to OGD as described (Goldberg and Choi, 1993). Briefly, the culture medium was replaced with a hypoxic medium previously saturated for 20 min with 95% N_2 and 5% CO_2 and containing 116mM NaCl, 5.4mM KCl, 0.8mM $MgSO_4$, 26.2mM $NaHCO_3$, 1mM NaH_2PO_4, 1.8mM $CaCl_2$, 0.01mM glycine and 0.001w/v phenol red . Hypoxic conditions were maintained using a hypoxia chamber (1 hour, 37°C, 95% N_2/5% CO_2). These experimental conditions induced a 30% pO_2 decrease in the medium.

Mitochondrial morphology analysis in primary neurons

To label mitochondria, neuronal cells were transfected with mitochondrially-targeted dsRED protein (mtRFP). Digital images were captured on a confocal microscope using a 63X oil immersion lens and, to compensate for optical imperfections of the microscope and to enhance the quality of images, subjected to a 2D deconvolution, using the ‘‘Integrative Deconvolution’’ plug-in for ImageJ software. Then, images were analyzed using the ImageJ macro "Morphometry" described in (Cribbs and Strack, 2009) to determine the two mitochondrial shape metrics form factor (FF) and aspect ratio (AR).

Cell death in primary cortical neurons

For TUNEL assays, primary neurons transfected and treated as indicated were seeded onto 13mm round glass coverslips, fixed, immunostained with anti-β-Tubulin III antibody (1:400, Sigma) and an isotype-matched Alexa-647 secondary antibody, and incubated in TUNEL reagent (Roche) following manufacturer’s instructions. Coverslips were mounted on slides with a DAPI-containing AntiFade ProGold mounting solution (Invitrogen). For DAPI, TUNEL, mtRFP and β-Tubulin III detection, coverslips were excited using the Diode 405/30 nm, Argon/2 488/514nm and HeNe 633nm lasers, respectively. Images were acquired sequentially using four separate color channels using of a Zeiss LSM Meta using a 63x Oil DIC EC Plan Neofluar objective (Zeiss). Cell death was measured as the percentage of TUNEL positive cells in the mtRPF/β-Tubulin III positive population. A total of 30 neurons were counted per condition in each independent experiment.

Supplemental References

Cribbs,J.T. and Strack,S. (2009). Functional characterization of phosphorylation sites in dynamin-related protein 1. Methods Enzymol. *457*, 231-253.

Goldberg,M.P. and Choi,D.W. (1993). Combined oxygen and glucose deprivation in cortical cell culture: calcium-dependent and calcium-independent mechanisms of neuronal injury. J. Neurosci. *13*, 3510-3524.

Perkins,D.N., Pappin,D.J.C., Creasy,D.M., and Cottrell,J.S. (1999). Probability-based protein identification by searching sequence databases using mass spectrometry data. Electrophoresis *20*, 3551-3567.
