# Supplementary material for: Extracellular Regulated Kinase Phosphorylates Mitofusin 1 to Control Mitochondrial Morphology and Apoptosis
Source: Mol Cell. 2015 Apr 16;58(2):244–54. doi: 10.1016/j.molcel.2015.02.021 (PMC4405354; doi:10.1016/j.molcel.2015.02.021)
Supplement: Document S2. Article plus Supplemental Information [file mmc2.pdf]

Molecular Cell

Article

Extracellular Regulated Kinase Phosphorylates Mitofusin 1 to Control Mitochondrial Morphology and Apoptosis

Graphical Abstract

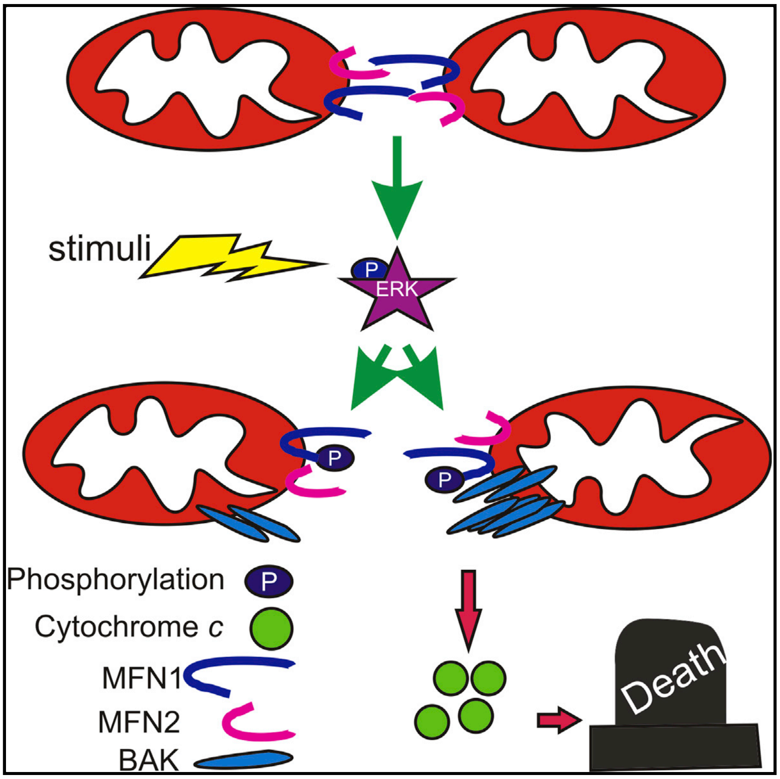

Highlights

- Mfn1 is phosphorylated by ERK to inhibit mitochondrial fusion
- Mfn1 phosphorylation stimulates mitochondrial permeabilization and apoptosis
- The MAPK cascade regulates mitochondrial shape and apoptosis via Mfn1

Authors

Aswin Pyakurel, Claudia Savoia, Daniel Hess, Luca Scorrano

Correspondence

luca.scorrano@unipd.it

In Brief

Pyakurel et al. show that the pleiotropic ERK kinase phosphorylates the mitochondrial fusion protein Mitofusin 1. Phosphorylation by ERK inhibits mitochondrial fusion and stimulates apoptotic mitochondrial permeabilization, identifying how mitochondrial shape and apoptosis

Pyakurel et al., 2015, Molecular Cell *58*, 244–254
April 16, 2015 ©2015 The Authors
http://dx.doi.org/10.1016/j.molcel.2015.02.021

Molecular Cell
Article

Extracellular Regulated Kinase Phosphorylates Mitofusin 1 to Control Mitochondrial Morphology and Apoptosis

Aswin Pyakurel,[1,2] Claudia Savoia,[1,2] Daniel Hess,[3] and Luca Scorrano[1,2,*]
[1]Dulbecco-Telethon Institute, Venetian Institute of Molecular Medicine, Via Orus 2, 35129 Padova, Italy
[2]Department of Biology, University of Padova, Via U. Bassi 58B, 35121 Padova, Italy
[3]Friedrich Miescher Institute for Biomedical Research, Maulbeerstrasse 66, 4058 Basel, Switzerland
*Correspondence: luca.scorrano@unipd.it
http://dx.doi.org/10.1016/j.molcel.2015.02.021
This is an open access article under the CC BY license (http://creativecommons.org/licenses/by/4.0/).

SUMMARY

Controlled changes in mitochondrial morphology participate in cellular signaling cascades. However, the molecular mechanisms modifying mitochondrial shape are largely unknown. Here we show that the mitogen-activated protein (MAP) kinase cascade member extracellular-signal-regulated kinase (ERK) phosphorylates the pro-fusion protein mitofusin (MFN) 1, modulating its participation in apoptosis and mitochondrial fusion. Phosphoproteomic and biochemical analyses revealed that MFN1 is phosphorylated at an atypical ERK site in its heptad repeat (HR) 1 domain. This site proved essential to mediate MFN1-dependent mitochondrial elongation and apoptosis regulation by the MEK/ERK cascade. A mutant mimicking constitutive MFN1 phosphorylation was less efficient in oligomerizing and mitochondria tethering but bound more avidly to the proapoptotic BCL-2 family member BAK, facilitating its activation and cell death. Moreover, neuronal apoptosis following oxygen glucose deprivation and MEK/ERK activation required an intact MFN1^{T562}. Our data identify MFN1 as an ERK target to modulate mitochondrial shape and apoptosis.

INTRODUCTION

Mitochondria sense and amplify apoptotic signals, releasing cytochrome *c* and other cofactors required for the activation of caspases and downstream cell death (Danial and Korsmeyer, 2004). Cytochrome *c* release requires mitochondrial outer membrane permeabilization (MOMP), is tightly regulated by proteins of the Bcl-2 family, and is associated with mitochondrial morphology changes (Frank et al., 2001). Mitochondrial morphology is modulated by large dynamin-like GTPases: the cytosolic dynamin-related protein 1 (DRP1), which translocates to mitochondria, binding to its receptor on the outer membrane (OMM) mitochondrial fission factor (MFF) (Otera et al., 2010), constricting and fragmenting mitochondria. Pro-fusion proteins display pleiotropic functions: the inner mitochondrial membrane (IMM) protein Optic Atrophy 1 (OPA1) not only promotes fusion (Cipolat et al., 2004), but it also regulates cristae shape and remodeling to control cytochrome *c* release (Frezza et al., 2006) and mitochondrial function (Cogliati et al., 2013). Of the two mammalian OMM mitofusins (MFNs) (Chen et al., 2003), MFN1 mediates organelle fusion together with OPA1 (Cipolat et al., 2004), whereas MFN2 tethers mitochondria either in *trans* (Koshiba et al., 2004) or to the endoplasmic reticulum (ER) (de Brito and Scorrano, 2008).

The role of mitochondrial morphology in cell death is not completely understood. Hyperfusion represents a recognized strategy to allow survival during nutrient deprivation and cellular stress (Gomes et al., 2011; Rambold et al., 2011; Tondera et al., 2009). Conversely, it has been questioned whether the fragmentation observed during apoptosis is causal for cytochrome *c* release (see, for example, Westermann, 2010 for a review). Several models have been proposed to explain why mitochondrial fragmentation facilitates apoptosis. Fragmentation has been associated with mitochondrial hemifusion driven by the pro-fission molecule DRP1 that stimulates BAX accumulation on mitochondria (Montessuit et al., 2010) or with the remodeling of mitochondrial cristae (Germain et al., 2005) that is required for maximal cytochrome *c* release (Scorrano et al., 2002). The observed fragmentation is the result of DRP1-mediated fission and of impaired mitochondrial fusion (Karbowski et al., 2004). Interestingly, mitochondrial fusion and the apoptotic machinery are molecularly linked, since the Bcl-2 family proapoptotic members BAX and BAK complex with MFNs to facilitate MFN2 oligomerization and activity in healthy cells (Karbowski et al., 2006). Conversely, during apoptosis, BAK dissociates from MFN2 and associates with MFN1 (Brooks et al., 2007), albeit the functional role and the molecular mechanism of this switch is not understood.

To understand how mitochondrial fusion changes during apoptosis, we tested whether key mitochondrial fusion molecules can be post-translationally modified. We show that MFN1 is phosphorylated at an atypical target site for the pleiotropic mitogen-activated protein (MAP) kinase cascade. This MFN1 phosphorylation modulates mitochondrial morphology, affecting its ability to form oligomers, and apoptosis, influencing BAK oligomerization in response to apoptotic stimuli.

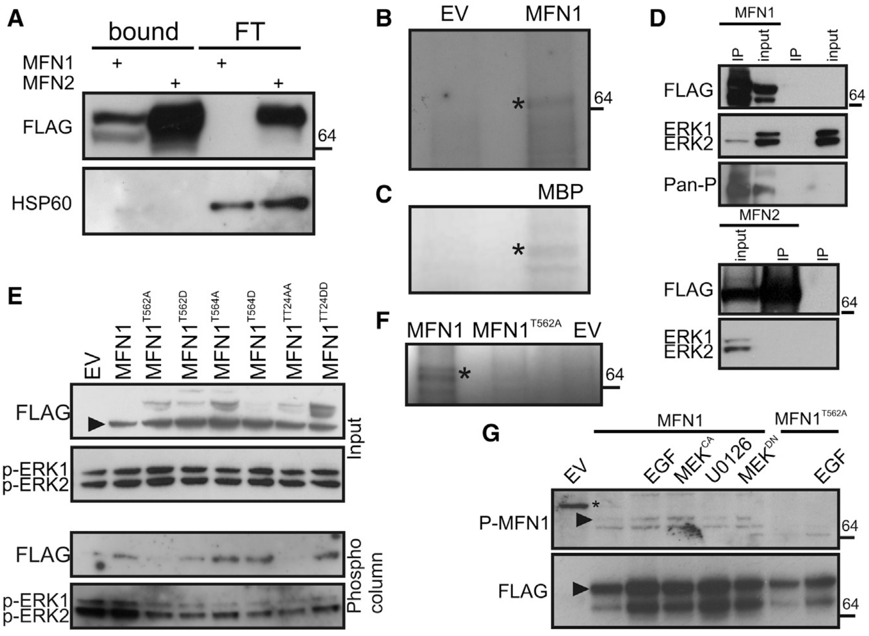

Figure 1. MFN1 Is Phosphorylated at T562

(A) Equal amounts of total lysates from *Mfn1*$^{-/-}$ or *Mfn2*$^{-/-}$ MEFs transfected, as indicated, were separated by phosphocolumn affinity chromatography. The indicated fractions were separated by SDS-PAGE and immunoblotted as indicated.

(B) The anti-FLAG immunoprecipitate from *Mfn1*$^{-/-}$ MEFs transfected, as indicated, was incubated with active recombinant ERK in a reaction buffer containing ^{32}P-labeled ATP. The mixture was separated by SDS-PAGE and transferred to a nitrocellulose membrane, and the radioactivity was detected. Asterisk: MFN1.

(C) Recombinant MBP was incubated with active recombinant ERK in a reaction buffer containing ^{32}P-labeled ATP. The mixture was separated by SDS-PAGE and transferred to a nitrocellulose membrane, and the radioactivity was detected. Asterisk: MBP.

(D) Where indicated (IP), FLAG-tagged proteins were immunoprecipitated from *Mfn1*$^{-/-}$ (top) or *Mfn2*$^{-/-}$ (bottom) MEFs transfected as indicated, separated by SDS-PAGE, and immunoblotted as indicated. Input was diluted 1:5 before loading.

(E) Equal amounts of total lysates from *Mfn1*$^{-/-}$ MEFs transfected as indicated were separated by phosphocolumn affinity chromatography, subjected to SDS-PAGE, and immunoblotted as indicated. Arrowhead: FLAG-MFN1.

(F) Experiment was as in (B).

(G) *Mfn1*$^{-/-}$ MEFs transfected as indicated were treated as indicated. FLAG-MFN1 was immunoprecipitated, separated by SDS-PAGE, and immunoblotted as indicated. Asterisk: unspecific band. Arrowhead: FLAG-MFN1.

See also Figure S1.

RESULTS

MFN1 Is Phosphorylated at T562

Whether and how phosphorylation regulates mitochondrial fusion is unclear: we therefore set out to identify if MFNs are phosphorylated. Phosphorylated and non-phosphorylated Flag-tagged MFN1 or MFN2 expressed in untreated mouse embryonic fibroblasts (MEFs) were separated by affinity chromatography on a column that specifically binds phosphorylated residues (Cereghetti et al., 2008). Immunoblotting indicated that both Flag-MFN1 and MFN2 avidly bound to the column (Figure 1A). To identify the phosphorylated site(s) of these proteins, large-scale anti-Flag immuno-purification from *Mfn1*$^{-/-}$ and *Mfn2*$^{-/-}$ cells expressing Flag-MFN1 and Flag-MFN2 was successfully carried out (Figure S1A), and the bands corresponding to MFNs were excised from the gel and analyzed by mass spectroscopy. While 83% of MFN1 sequence was covered, coverage for MFN2 was only around 65% (Figure S1C), possibly explaining why we could not detect any MFN2 phosphorylation. Conversely, T562 and possibly T564 that lie in the HR1 region of MFN1 were found to be phosphorylated (Figure S1B). The sequence of this region, ASTPTAP, resembles a canonical ERK site, Pro-Xaa-Ser/Thr-Pro (X = neutral/basic amino acid) (Clark-Lewis et al., 1991), suggesting that this could be an atypical ERK site. Indeed, an in vitro phosphorylation assay using active recombinant ERK on immunoprecipitated FLAG-MFN1 and ^{32}P-labeled ATP indicated that ERK can directly phosphorylate MFN1 (Figure 1B) like its canonical target myelin basic protein (MBP) (Figure 1C). Moreover, that MFN1 can be an ERK target was further substantiated by the finding that ERK co-immunoprecipitated with FLAG-MFN1 but not FLAG-MFN2 (Figure 1D).

To discriminate between the two identified sites of phosphorylation, we first analyzed them in silico. T562 is conserved throughout mammalian MFN1s (Figure S1E). Of note, this residue is conserved also in MFN2, but the phosphoproteomic data excluded its phosphorylation, (data not shown) and MFN2 did not bind to ERK (Figure 1D). We next generated single and double mutants mimicking phosphorylated (MFN1^{T562D}, MFN1^{T564D}, and MFN1^{T562D,T564D}) or dephosphorylated MFN1 (MFN1^{T562A}, MFN1^{T564A}, and MFN1^{T562A,T564A}). Upon phosphocolumn affinity purification of the expressed mutants, only MFN1^{T562A} did not bind to the substrate, whereas all the T564 mutants showed comparable binding (Figure 1E). In the same in vitro phosphorylation assay used in Figure 1B, recombinant ERK was unable to phosphorylate the MFN1^{T562A} mutant (Figure 1F), further indicating that T562 could be the residue phosphorylated by ERK. To unequivocally prove that ERK phosphorylate MFN1^{T562}, we produced a specific antibody directed to pT562 of MFN1. This antibody immunodecorated wild-type (wt), but not the T562A MFN1 mutant re-expressed in *Mfn1*$^{-/-}$ MEFs (Figure 1G, arrowhead), lending strong support to the fact that MFN1 was phosphorylated by ERK at T562. Moreover, treatment of cells with epidermal growth factor (EGF) that activates MEK and ERK, as well as genetic ERK activation (Figure S3) by a constitutively active MEK (MEK^{CA}) (Pagès et al., 1994), increased T562 phosphorylation levels; pharmacological or genetic MEK inhibition using the selective U0126 inhibitor or by expressing a dominant negative MEK mutant (MEK^{DN}); and

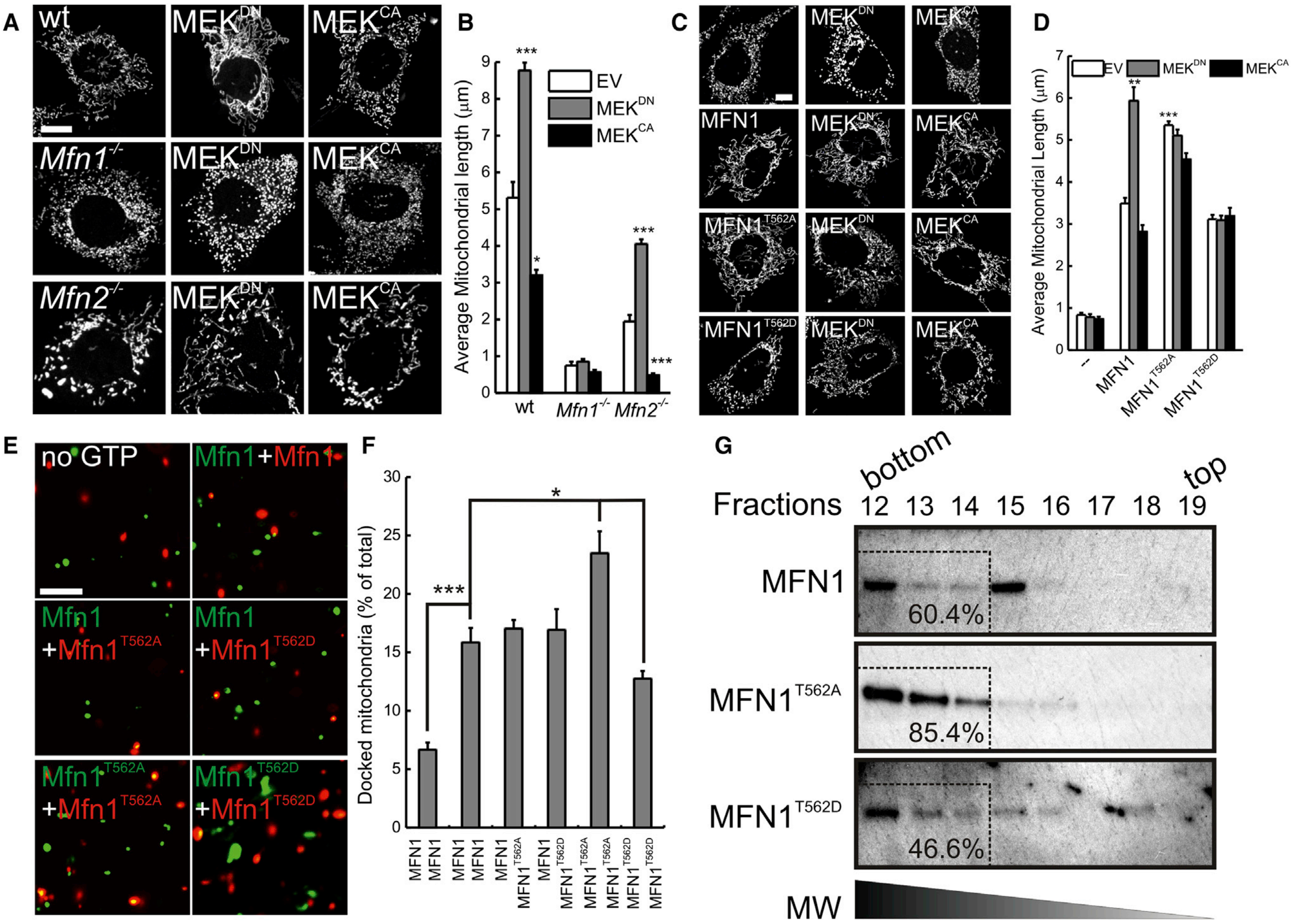

Figure 2. ERK Modulates Mitochondrial Morphology via MFN1^{T562}

(A) Representative confocal images of mitochondrial morphology in MEFs of the indicated genotype cotransfected with mtYFP and indicated plasmids. Scale bar, 10 μm.

(B) Experiments were carried out as in (A). Data represent mean ± SEM of five independent experiments (n = 30 cells per condition). *p < 0.05; **p < 0.01; ***p < 0.001 versus EV.

(C) Representative confocal images of mitochondrial morphology in *Mfn1*$^{-/-}$ cotransfected with mtYFP and the indicated plasmids. Scale bar, 10 μm.

(D) Experiments were carried out as in (C). Data represent mean ± SEM of five independent experiments (n = 30 cells per condition). *p < 0.05; **p < 0.01; ***p < 0.001 versus MFN1+EV.

(E) Representative confocal images of mitochondrial binding assay in mitochondria isolated from *Mfn1*$^{-/-}$ cells reconstituted and transfected as indicated. Scale bar, 1 μm.

(F) Experiments were carried out as in (D). Data represent mean ± SEM of three independent experiments. *p < 0.05; ***p < 0.001.

(G) Mitochondria from *Mfn1*$^{-/-}$ MEFs reconstituted as indicated were homotypically mixed and dissolved in 1% digitonin. Complexes were separated according to their MW by sucrose-gradient centrifugation and equal amounts of proteins from each fraction were subjected to SDS-PAGE and immunoblotted as indicated. The percentage of MFN1 in the boxed high-MW fractions over the total MFN1 retrieved is indicated.

See also Figure S2.

reduced T562 phosphorylation; finally, in *Mfn1*$^{-/-}$ cells re-expressing the MFN1^{T562A} mutant, phosphorylation was undetectable even in the presence of EGF (Figure 1G). Taken together, these data indicate that ERK phosphorylates MFN1 at T562.

ERK Modulates Mitochondrial Morphology via MFN1^{T562}

We next explored the significance of ERK-mediated MFN1 phosphorylation in mitochondrial morphology. Expression of MEK^{DN} caused mitochondrial elongation, whereas activation of ERK by MEK^{CA} resulted in fragmentation (Figures 2A and 2B). Furthermore, in cells lacking *Mek1* or *Mek2* mitochondria were significantly elongated (Figures S2A and S2B). As the MEK mutants did not modify mitochondrial morphology in *Mfn1*$^{-/-}$ MEFs, we concluded that MEK requires MFN1 to modulate mitochondrial shape (Figures 2A and 2B), and we set out to investigate whether it depended on MFN1^{T562}. As expected, MEK^{DN} triggered mitochondrial elongation while MEK^{CA} fragmentation in *Mfn1*$^{-/-}$ MEFs re-expressing wt MFN1. However, MEK^{DN} and MEK^{CA} were ineffective in *Mfn1*$^{-/-}$ MEFs reconstituted with the non-phosporylable MFN1^{T562A} and MFN1^{T562D} mutants, where

mitochondria were constitutively elongated or fragmented (Figures 2C and 2D). These experiments indicate that ERK modulates mitochondrial morphology by impinging on MFN1^{T562}. Since MFN1 is crucial in *trans* for mitochondrial docking, the initial step of mitochondrial fusion (Ishihara et al., 2004; Koshiba et al., 2004), we set out to understand the mechanism by which MFN1^{T562} phosphorylation impinges on mitochondrial morphology by turning to an established assay of mitochondrial tethering (Ishihara et al., 2004). We therefore mixed mtGFP- or mtRFP-labeled mitochondria isolated from cells stably expressing wt and/or mutated MFN1, imaged them by confocal microscopy, and scored the images for the appearance of yellow mitochondria indicative of tethering of individually labeled organelles (Ishihara et al., 2004). GTP-stimulated tethering of MFN1-MFN1 mitochondria was superimposable to that recorded with the MFN1-MFN1^{T562A} or MFN1-MFN1^{T562D} pairs. Conversely, tethering of MFN1^{T562D}-MFN1^{T562D} mitochondria was significantly reduced, whereas that of MFN1^{T562A}-MFN1^{T562A} mitochondria was increased (Figures 2E and 2F). During the docking reactions primed by GTP, MFN1 assembles oligomers at lower M.W. formed in *cis* as well as in a "docking" higher M.W. (~450 kDa) complex (Ishihara et al., 2004). Upon sucrose gradient differential centrifugation of digitonin solubilized membranes from homotipically mixed, GTP primed *Mfn1*$^{-/-}$ mitochondria reconstituted with MFN1, MFN1^{T562A} or MFN1^{T562D}, MFN1^{T562A}, and MFN1 were retrieved mostly in the docking, high-molecular weight (MW) oligomer. Conversely, MFN1^{T562D} was mostly recovered in lower MW oligomers (fractions 18 and 19), where wt MFN1 and MFN1^{T562A} are not found (Figure 2G). Thus, MFN1^{T562} regulates MFN1 oligomerization and hence mitochondrial tethering and fusion.

Phosphorylation of Mfn1 by ERK Regulates Cell Death

Since both the MEK/ERK pathway and changes in mitochondrial morphology have been implicated in the modulation of apoptosis (Green and Kroemer, 2004; McCubrey et al., 2007; Westermann, 2010), we wondered whether the MFN1 phosphorylation identified here could link the two pathways in cell death. When we measured apoptosis in cells lacking *Mek1* or *Mek2*, we found that cell death was significantly reduced (Figure S3A). Indeed, MFN1^{T562} phosphorylation was increased in cells treated with the mitochondria-dependent intrinsic death stimulus etoposide (Figure S3B), and expression of MEK^{DN} that, as expected, inhibited ERK phosphorylation (Figure S3C) delayed apoptosis not only by etoposide but also by the other intrinsic stimuli staurosporine and H_2O_2 in wt and *Mfn2*$^{-/-}$, but not in *Mfn1*$^{-/-}$ MEFs (Figure 3A). Conversely, increased apoptosis was measured in Wt and *Mfn2*$^{-/-}$, but not *Mfn1*$^{-/-}$ cells expressing MEK^{CA} (Figure S3D). The finding that cytochrome *c* release by H_2O_2 was blunted by MEK^{DN} in wt and *Mfn2*$^{-/-}$ cells, but not in *Mfn1*$^{-/-}$ MEFs (Figures 3B and 3C) further substantiated that ERK requires MFN1 to modulate apoptosis at the mitochondrial level. Cytochrome *c* release requires OMM permeabilization, effected by the multidomain proapoptotics Bcl-2 family members BAX and BAK (Wei et al., 2001). BAK oligomerization in response to recombinant caspases-8-cleaved BID (cBID) was increased in mitochondria from cells where ERK was activated by EGF treatment (Figures 3D–3F). Conversely in *Mek1*$^{-/-}$ mitochondria cBID-induced BAK oligomerization was reduced (Figure S3E), confirming a link between the MEK/ERK pathway and BAK activation, whereas MEK^{DN} expression did not change H_2O_2-induced BAX activation in all the cell lines tested (Figure S4). We therefore decided to further explore how MFN1^{T562} modulated BAK activation. We first verified that in *Mfn1*$^{-/-}$ mitochondria reconstituted with phosphomimetic MFN1^{T562D} cBID-induced BAK oligomerization was increased (Figures 4A and 4B). To directly verify if the increased BAK activation was operated by ERK, we turned to *Mfn1*$^{-/-}$ cells reconstituted with MFN1 or with the non-phosphorylable mutant MFN1^{T562A} that per se does not affect BAK oligomerization (Figure 4B). While EGF treatment increased BAK oligomerization and cytochrome c release in *Mfn1*$^{-/-}$::MFN1 cells, it was ineffective in *Mfn1*$^{-/-}$::MFN1^{T562A} cells (Figures 4C–4E). Along the same line, *Mfn1*$^{-/-}$::MFN1^{T562A} MEFs were less, while *Mfn1*$^{-/-}$::MFN1^{T562D} were more, susceptible to staurosporine. Both *Mfn1*$^{-/-}$::MFN1^{T562A} and *Mfn1*$^{-/-}$::MFN1^{T562D} MEFs were insensitive to MEK mutants expression (Figure 4F). Finally, in a pulldown experiment MFN1^{T562D} bound more BAK than wt and MFN1^{T562A} (Figure 4G), suggesting that T562 phosphorylation regulates MFN1-BAK binding and activation of the latter. In conclusion, ERK-phosphorylated MFN1 favors cytochrome *c* release by increasing BAK oligomerization.

MFN1 Phosphorylation Regulates Apoptosis in Primary Cortical Neurons

To better understand the physiological relevance of MEK/ERK modulation of mitochondrial morphology and permeabilization, we turned our attention to oxidative stress, during which MEK/ERK are activated. Indeed, H_2O_2 induced rapid ERK phosphorylation in MEFs (data not shown) before the observed mitochondrial fragmentation, which was counteracted by the expression of a non-phosphorylable MFN1^{T562A} mutant (Figures S5A and S5B). Comforted by these results, we turned our attention to oxygen glucose deprivation (OGD), an established in vitro model of ischemic damage where the MEK/ERK pathway is activated (Yung and Tolkovsky, 2003) and contributes to primary cortical neurons damage (Gao et al., 2014; Stanciu et al., 2000). First we addressed if the MEK/ERK pathway and MFN1^{T562} participated in the regulation of mitochondrial morphology in neurons. Mitochondria appeared as short rods in the soma and in the neurites of primary cortical neurons. Enforced MFN1 expression triggered mitochondrial elongation, leading to an intricate network of long organelles in both soma and neurites. EGF treatment resulted in mitochondrial fragmentation in both soma and neurite. Conversely, in neurons expressing the non-phosphorylable MFN1^{T562A} mutant, upon EGF treatment the mitochondria remained elongated and evenly distributed (Figures S5C and S5D), suggesting that also in neurons ERK activation impinges on mitochondria via MFN1^{T562} phosphorylation. We next verified whether this pathway was operational during OGD. Mitochondria appeared elongated and retained cytochrome c in healthy β-tubulin III-positive neurons from primary cortical cultures (top panels in Figures 5A and 5B). During OGD, the observed massive mitochondrial fragmentation was accompanied by cytochrome c release and followed by apoptosis, as indicated by the neuronal TUNEL positivity. MFN1 expression inhibited the mitochondrial phenotypes and cell death (bottom panels in Figures 5A and 5B and quantification in Figures 5C–5E). Of note, while EGF

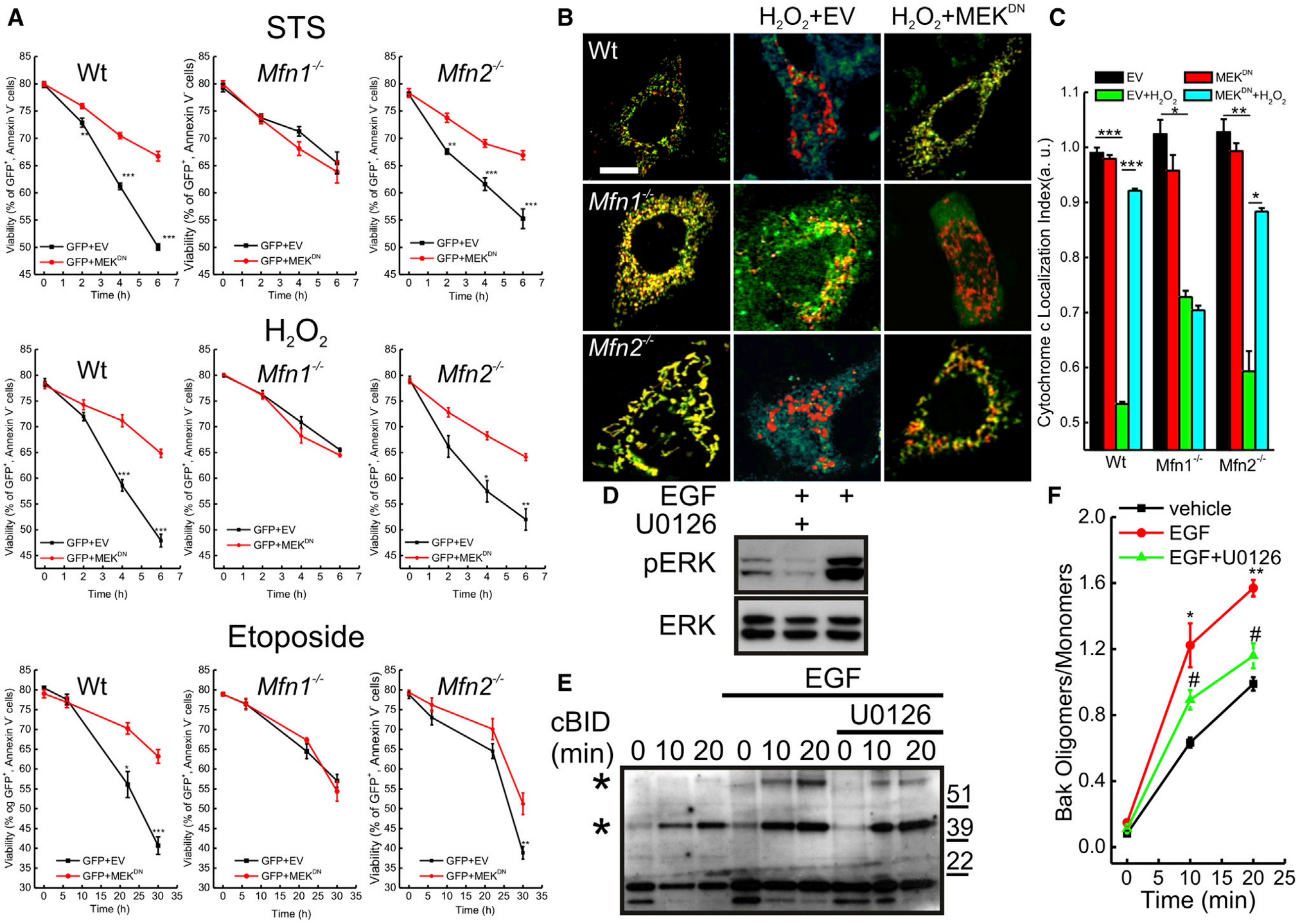

Figure 3. Phosphorylation of MFN1 by ERK Modulates Cell Death

(A) MEFs of indicated genotypes cotransfected with GFP, and the indicated plasmids were treated with 2 μM staurosporine, 1 mM H_2O_2, or 2 μM etoposide for the indicated times. Data are mean ± SEM of five independent experiments. *p < 0.05; **p < 0.01; ***p < 0.001 versus GFP +EV.

(B) Representative confocal images of subcellular cytochrome *c* (green) distribution in MEFs of indicated genotypes cotransfected with mtRFP (red) and the indicated plasmids. Where indicated, cells were treated for 30 min with 1 mM H_2O_2. Scale Bar, 10 μm.

(C) Localization index of cytochrome *c*. Experiments were performed as in (B). Data represent mean ± SEM of three independent experiments. *p < 0.05, **p < 0.01, ***p < 0.001.

(D) Equal amounts (30 μg) of proteins from total lysates of MEFs treated where indicated with U0126 (10 μM) for 1 hr and EGF (10 nM) for 10 min were analyzed by SDS-PAGE/immunoblotting.

(E) Mitochondria isolated from MEFs treated where indicated with 10 nM EGF were treated with cBID for the indicated times and crosslinked with 10 mM BMH. Equal amounts (30 μg) of proteins were analyzed by SDS-PAGE/immunoblotting. Asterisks: BAK multimers.

(F) Densitometric analysis of BAK oligomerization. Data represent mean ± SEM of three independent experiments. *p < 0.05; **p < 0.01 (versus untreated); $^{\#}p$ < 0.05 (versus EGF).

See also Figure S3.

treatment abolished the MFN1 protective effect, it did not affect neurons expressing the non-phosphorylable MFN1^{T562A} mutant (Figure 5), suggesting that active ERK impinges on MFN1^{T562} also during neuronal OGD. Our results indicate that MFN1 phosphorylation participates in relevant models of neuronal death where the MEK/ERK pathways are turned on.

DISCUSSION

Here we demonstrate that the outer mitochondrial membrane fusion protein MFN1 is phosphorylated by ERK to regulate mitochondrial morphology and apoptotic permeabilization. Our results indicate that when MFN1 is phosphorylated by ERK, mitochondria fragment and BAK oligomerization is favored, increasing susceptibility to apoptotic stimuli.

Considering that cytosolic DRP1 needs signals to translocate, form oligomers, and constrict mitochondria, it is not surprising that post-translation modifications including phosphorylation at different sites and SUMOylation tightly regulate it (Otera et al., 2013). Less is known on how components of the mitochondrial fusion machinery respond to cellular cues. OPA1 that sits at the interface between mitochondrial fusion and apoptosis, regulating fusion (Cipolat et al., 2004) as well as cristae shape and remodeling (Frezza et al., 2006), is post-transcriptionally cleaved

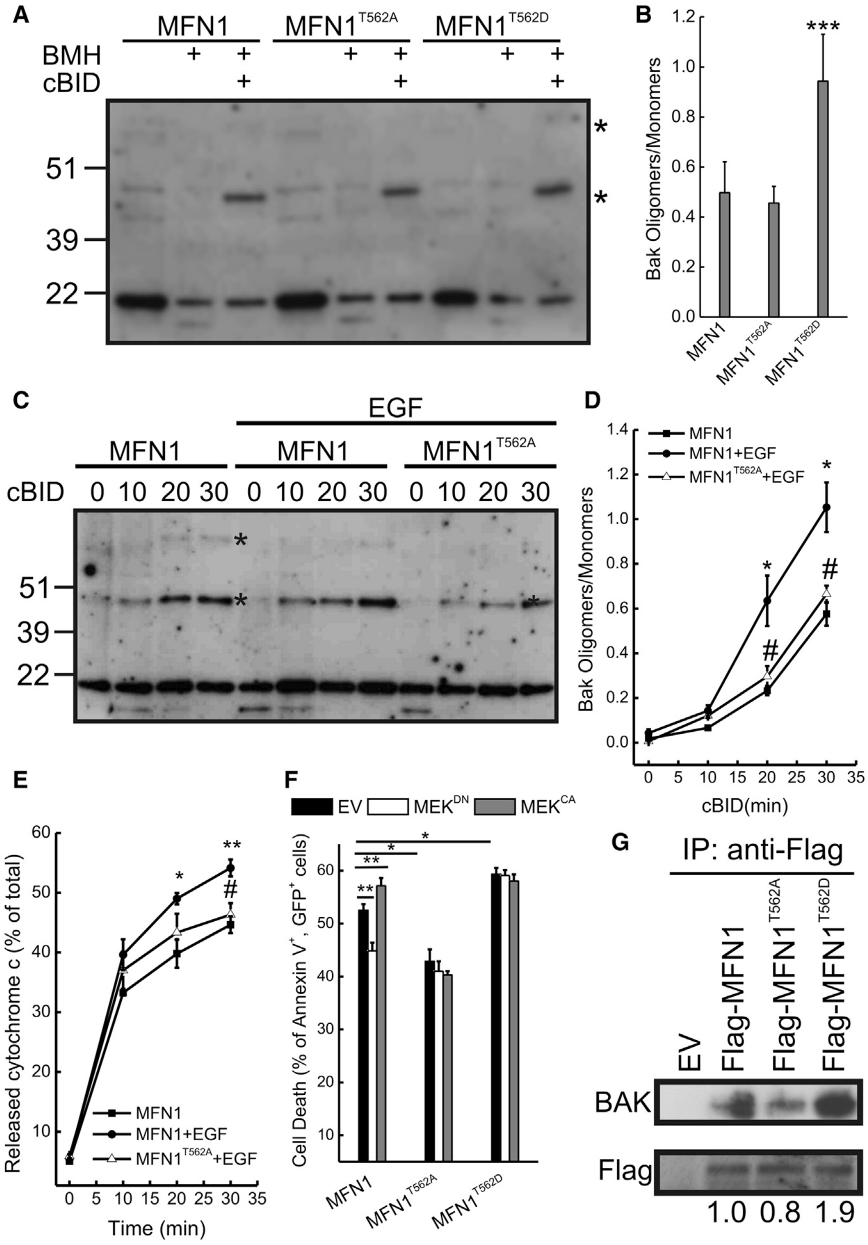

Figure 4. MFN1 Phosphorylation Modulates BAK Oligomerization

(A) Mitochondria from *Mfn1*$^{-/-}$ MEFs stably expressing the indicated MFN1 were treated with cBID as indicated and crosslinked with 10 mM BMH as indicated. Equal amounts (30 μg) of proteins were analyzed by SDS-PAGE/immunoblotting. Asterisks: BAK multimers.

(B) Densitometric analysis of BAK oligomerization. Data represent average ± SEM of four independent experiments. ***$p < 0.001$ versus MFN1.

(C) Mitochondria isolated from *Mfn1*$^{-/-}$ MEFs stably expressing the indicated MFN1 treated where indicated with EGF (10 nM for 10 min) were treated where indicated with cBID and crosslinked with 10 mM BMH. Equal amounts (30 μg) of proteins were analyzed by SDS-PAGE/immunoblotting. Asterisks: BAK multimers.

(D) Densitometric analysis of BAK oligomerization. Data represent average ± SEM of four independent experiments. *$p < 0.05$ (versus MFN1); $^{\#}p < 0.05$ (versus MFN1+EGF).

(E) Mitochondria isolated from *Mfn1*$^{-/-}$ MEFs stably expressing the indicated MFN1 treated where indicated with EGF (10 nM for 10 min) were treated with cBID for the indicated times, and cytochrome *c* release was measured. Data represent average ± SEM of four independent experiments. *$p < 0.05$; **$p < 0.01$ (versus MFN1); $^{\#}p < 0.05$ (versus MFN1+EGF).

(F) *Mfn1*$^{-/-}$ MEFs cotransfected with GFP and the indicated plasmids were treated with 2 μM staurosporine for 6 hr. Data represent average ± SEM of four independent experiments. *$p < 0.05$; **$p < 0.01$.

(G) FLAG-MFN1 was immunoprecipitated from *Mfn1*$^{-/-}$ MEFs transfected as indicated and analyzed by SDS-PAGE/immunoblotting as indicated.

See also Figure S4

by different proteases that produce short versions required for its biological activities (Meeusen et al., 1999). Conversely, post-translational modifications in MFNs are hard to retrieve: given their membrane localization, they are difficult to immunopurify and therefore to analyze by mass spectrometry. Despite these difficulties, MFNs have been found to be ubiquitinated, even if the exact site of ubiquitination has not yet been identified. Interestingly, Mfn is a substrate of the E3 ubiquitin ligase Parkin, mutated in Parkinson's disease (PD) (Ziviani et al., 2010), and Parkin mutations associated with genetic forms of PD compromise the ability to ubiquitinate Mfn (Glauser et al., 2011), implying Mfns in the function of dopaminergic neurons and in pathogenesis of PD. More recently, MFN2 was also found to be phosphorylated: Pink1 phosphorylates MFN2 to convert it into a mitochondrial receptor for Parkin (Chen and Dorn, 2013) and stress-induced phosphorylation by JNK causes MFN2 degradation through the ubiquitin-proteasome pathway (Leboucher et al., 2012). However, whether the core mitochondrial fusion protein MFN1 is also post-translationally modified was unclear. Large-scale phosphorylation analysis of whole organs in mouse identified S590 as a site of MFN1 phosphorylation (Beausoleil et al., 2006). However, phosphorylation of this site was restricted to kidney and lung, where the same site in MFN2 was also found to be phosphorylated, suggesting that S590 could be a tissue-specific regulator of mitochondrial OMM fusion. Conversely, our results indicate that MFN1 (and not MFN2) is specifically phosphorylated by the MEK/ERK pathway, suggesting that mitochondrial morphology is a previously unidentified target of this pleiotropic regulatory signaling cascade (Shaul and Seger, 2007). Indeed, the OMM localization of MFNs appear ideal to relay cellular cues to mitochondria: the MFNs two heptad repeat (HR) and GTPase domains are exposed

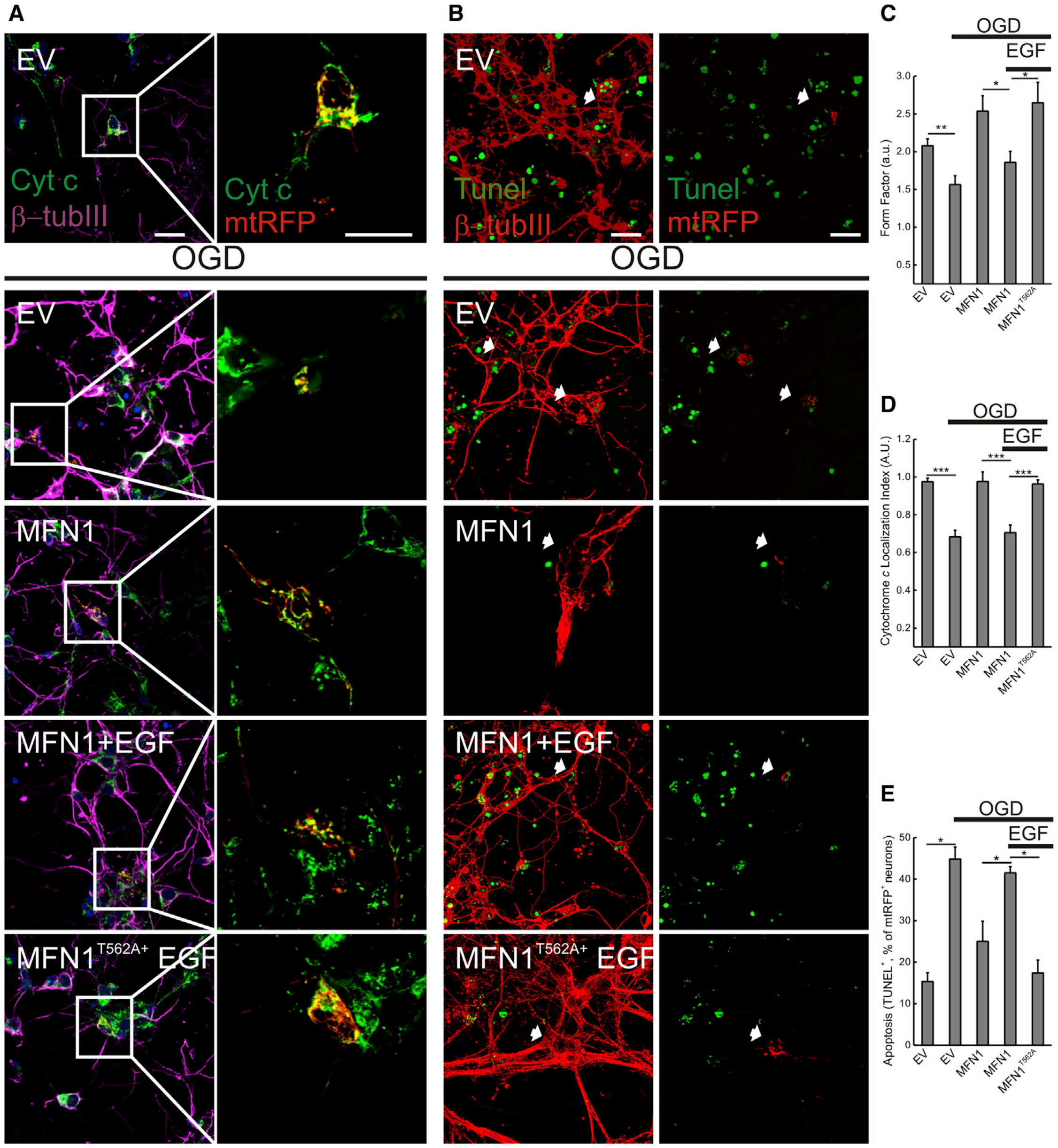

Figure 5. MFN1 Phosphorylation Regulates Mitochondrial Morphology and Cell Death in Primary Cortical Neurons

(A) Representative images of primary cortical neurons co-transfected with mtRFP (red) and the indicated plasmids and treated with EGF where indicated. If indicated, cells were exposed to OGD for 1 hr. After fixation, cells were immunostained for cytochrome *c* (green) and β-tubulin III (purple). For the sake of clarity the mtRFP channel was omitted from the left merged image. Scale bar, 20 μm.

(B) Representative images of primary cortical neurons co-transfected with mtRFP and the indicated plasmids and treated with EGF where indicated, fixed, and stained for TUNEL (green) and β-tubulin III (red). If indicated, cells were exposed to OGD for 1 hr. For the sake of clarity, TUNEL staining (Green) has been merged with β-tubulin (red, left panel) and TUNEL (Green) with mtRFP (red, right panel). Arrow indicates TUNEL-positive transfected cells. Scale bar, 20 μm.

(C) Quantification of mitochondrial morphology. Experiments were performed as in (A). Data represent mean ± SEM of four independent experiments. *$p < 0.05$; **$p < 0.01$.

(D) Localization index of cytochrome *c*. Experiments were performed as in (A). Data represent mean ± SEM of four independent experiments. ***$p < 0.001$.

(E) Quantification of apoptosis. Experiments were performed as in (B). Data represent mean ± SEM of four independent experiments. *$p < 0.05$.

See also Figure S5.

to the cytosolic signaling cascades, making mitochondrial fusion an attractive mechanism to regulate organelle shape, localization, and physiology in response to intra- or extra-cellular stimuli (Campello and Scorrano, 2010). Mechanistically, antiparallel interactions of MFN1 HR2 domains in *trans* constitute the basis for mitochondrial tethering (Koshiba et al., 2004). The

phosphorylation site identified here is located; however, in the HR1 region and its mutation affects the oligomerization and docking ability of MFN1, raising the possibility, not investigated by Koshiba and colleagues, that also HR1 is crucial for MFN1-dependent mitochondrial tethering. During cellular stress, docking-competent MFN1 oligomers are reduced, thereby impairing mitochondrial tethering and fusion. Of note, the same residue is conserved in MFN2 (where the sequence is closer to a canonical ERK phosphorylation site), but there it was not retrieved to be phosphorylated. If the lack of phosphorylation is determined by different topology or subcellular localization of Mfn2 is unknown, but worth testing, especially in the light of the functional difference between the two Mfns.

While DRP1 post-translational modifications are well established players in apoptosis (Cribbs and Strack, 2007), necrosis (Wang et al., 2012), and autophagy (Gomes et al., 2011), our knowledge of how mitochondrial fusion is modulated during cell death is scarcer. Fusion inhibition is an established consequence of apoptosis induction, and it occurs around the time of BAX activation (Karbowski et al., 2004). However, how fusion is blocked is not fully understood; OMM permeabilization with the release of the soluble fraction of OPA1 (Arnoult et al., 2005b; Arnoult et al., 2005a) and the proteolytic cleavage of the long OPA1 forms (DeVay et al., 2009) as a consequence of mitochondrial dysfunction (Duvezin-Caubet et al., 2006) could explain mitochondrial fusion inhibition only during late apoptosis. Proapoptotic BCL-2 family members appear to participate in the earlier inhibition of fusion, occurring before or around the activation of BAX. While at rest, BAK and BAX facilitate MFN2 assembly, promoting its activity (Karbowski et al., 2006); during apoptosis, BAK dissociates from MFN2 and associates with MFN1 (Brooks et al., 2007). Our results suggest that this increased BAK-MFN1 association could depend on the phosphorylation status of the latter. In fact, the Mfn1^{T562D} mutant that mimics MFN1 phosphorylation by ERK binds more avidly to BAK, favoring its oligomerization, and causes mitochondrial fragmentation, perhaps by "stealing" MFN1 from the fusion reaction. Similarly EGF, which activates ERK, increases BAK oligomerization in a Mfn1^{T562}-dependent manner, placing MFN1 at the crossroad between extracellular signals and activation of the mitochondrial pathway of apoptosis.

During apoptosis, blockage of DRP1-dependent fragmentation is protective (Frank et al., 2001; Cereghetti et al., 2010). In addition to DRP1, our data indicate a role for the ERK-mediated inhibition of fusion in mitochondrial fragmentation. During apoptosis induced by the intrinsic stimuli tested here, ERK is phosphorylated (not shown), as previously reported in the case of oxidative stress (Kodiha et al., 2009), but its role during apoptosis remains unclear (Rasola et al., 2010). Our model indicates that the activation of MEK/ERK inhibits the pro-fusion function of MFN1 and triggers its association with BAK, facilitating its oligomerization and therefore cytochrome *c* release and cell death. Our data therefore place MFN1 in the core apoptotic pathway of OMM permeabilization. Whether phosphorylated MFN1 facilitates the dissociation of BAK from BCL-2, or it shifts the equilibrium of the BH3-only BAK reaction toward their interaction, remains to be clarified.

The effect of phosphorylated MFN1 extended beyond cellular stress. Since H_2O_2-induced mitochondrial fragmentation requires MFN1 phosphorylation, we investigated oxygen-glucose deprivation in primary cortical neurons, a condition mimicking disease-associated tissue damage and pathological ROS production. Our findings that MFN1 phosphorylation is involved in the cellular and neuronal response to oxidative stress and OGD extend the role of this modification to a relevant pathological condition. Finally, our results provide a conceptual framework for the analysis of mitochondrial fusion in the many kinds of cancer where the MEK/ERK pathway is constitutively upregulated (Dhillon et al., 2007).

EXPERIMENTAL PROCEDURES

Molecular Biology

FLAG-MFN1 and FLAG-MFN2 plasmids were a gift from Dr. Yuka Eura (Kyushu University). PBabe-MEK^{DN} and PBabe -MEK^{CA} were a gift from Prof. Chris Marshall (Institute of Cancer Research, London, UK). Amphotrophic viruses were generated by co-transfecting the HEK293-packaging cell lines with the packaging vector pIK and the required pMIG constructs as previously described (Cheng et al., 2001). Viral supernatants were retrieved and used to transduce MEFs in the presence of 4 μg/ml of hexadimethrine bromide (Sigma). Following an overnight transduction, cells were selected on 5 μg/ml puromycin or 400 μg/ml hygromycin. Details on the primers used to generate FLAG-MFN1 mutants by site directed mutagenesis can be found in the Supplemental Information.

Cell Culture

Simian virus 40 (SV40)-transformed wild-type, *Mfn1*$^{-/-}$, and *Mfn2*$^{-/-}$ MEFs were from D. Chan (California Institute of Technology) and cultured as described previously (de Brito and Scorrano, 2008). Wild-type and *Bax*$^{-/-}$*Bak*$^{-/-}$ MEFs were cultured as described previously (Scorrano et al., 2003). Transfection of MEFs with DNA was carried out using Transfectin (Biorad) according to the manufacturer's instructions. When indicated, cells were transfected 24 hr after plating.

Primary cultures of cortical neurons from P0 Wistar rats were prepared by modifying a previously described method (Abramov et al., 2007). The brain tissue was minced and trypsinized (0.1% for 15 min at 37°C), triturated, plated on poly-ornithine-coated coverslips, and cultured in Neurobasal-A medium (Invitrogen) supplemented with B-27 (Invitrogen) and L-glutamine (2 mM). For immunocytochemistry experiments, 1 × 10^5 neurons were plated on 13-mm glass coverslips. Cultures were maintained at 37°C in a humidified atmosphere of 5% CO_2 and 95% air, fed twice a week, and maintained for a minimum of 10 days before use. The procedures for OGD, mitochondrial morphology analysis, and TUNEL staining can be found in Supplemental Information.

Analysis of Cell Death

MEFs treated as indicated were stained with propidium iodide (PI) and Annexin-V-FITC (Bender MedSystem). Where indicated, cells were cotransfected with pEGFP and the indicated vector. After 24 hr cells were treated as described and stained with Annexin-V-PE (Bender MedSystem) according to the manufacturer's protocol. Cell death was measured by flow cytometry (FACSCalibur) as the percentage of Annexin-V-positive events in the GFP-positive population and viability as the percentage of Annexin-V-negative, PI-negative cells for transfected and untransfected cells, respectively.

Immunoprecipitation and Phosphoprotein Purification

For immunoprecipitation experiments, cells were lysed in CPBS buffer (6 mM CHAPS in PBS [pH 7.4]). Lysates were incubated with the indicated antibody (1:50, 14 hr, 4°C), and the protein-antibody complex was precipitated by centrifugation after incubation with protein G-coated magnetic beads (Dynal, 2 hr, 4°C). The immunoprecipitated material was washed twice in CPBS and resuspended in SDS/PAGE loading buffer (NuPAGE), boiled, and loaded on 4%–12% gels (NuPAGE). For phosphorylation studies, total cell lysates were loaded on a phosphoprotein binding column (QIAGEN) as previously described (Cereghetti et al., 2008). Flow-through (unphosphorylated) and

eluted (phosphorylated) proteins were collected and concentrated and 20 μg of proteins were separated by 4%–12% SDS-PAGE.

Mass Spectrometric Analysis

Cell extracts (10 mg) from *Mfn1*$^{-/-}$ and *Mfn2*$^{-/-}$ MEFs transfected with Flag-MFN1 and Flag-MFN2, respectively, were immunoprecipitated using anti-Flag antibody. The immunoprecipitates were separated by 4%–12% Tris-MOPS SDS-PAGE (NuPage, Invitrogen) and stained with Simply Blue Safe Stain (Invitrogen). Bands corresponding to Flag-MFN1 and Flag-MFN2 were excised and reduced with TCEP, alkylated with iodoacetamide and digested with trypsin or AspN. Details on liquid chromatography-mass spectrometry analysis can be found in Supplemental Information.

Biochemistry

Mitochondria were isolated by standard differential centrifugation in isolation buffer (IB) as described in Frezza et al. (2007). For BAK oligomerization assay, mitochondria (0.5 mg/ml) were incubated in experimental buffer (EB: 125 mM KCl, 10 mM Tris-MOPS [pH 7.4], 1 mM Pi, 5 mM glutamate, 2.5 mM malate, and 10 μM EGTA-Tris [pH 7.4]), and treated as described at 25°C. At the indicated time, mitochondria were pelleted by centrifugation at 12,000 × *g* at 4°C for 3 min and resuspended in the same volume of EB. For crosslinking, mitochondria were incubated with 1 mM (30 min, 37°C) BMH (Pierce), and the reaction was quenched with 20 mM β-mercaptoethanol (15 min, 25°C).

Sucrose-gradient separation of MFN1-containing complex was carried out following Ishihara et al. (2004). Mitochondria isolated from *Mfn1*$^{-/-}$ MEFs reconstituted as indicated were incubated in EB (0.5 mg/ml) containing 1 mM GTP for 30 min at 25°C and solubilized with 1% digitonin for 30 min at 4°C. A discontinuous sucrose density gradient containing 5%–20% sucrose solutions was prepared by layering successive decreasing sucrose densities solutions (5%, 7.5%, 10%, 12.5%, 15%, 17.5%, and 20%) upon one another. The cleared mitochondrial lysate was layered on 7 ml of the sucrose gradient and centrifuged at 174,000 × *g* for 12 hr at 4°C (Beckman, 100Ti). The centrifuged solution was separated into 22 fractions, and which fractions MFN1 eluted in were checked. After confirming the elution of MFN1 in fractions 12–19, these fractions were immunoblotted using the indicated antibodies.

For immunoblotting, proteins were separated by 7% Tris-acetate, 3%–8% Tris-acetate or 4%–12% Tris-MOPS SDS-PAGE (NuPage, Invitrogen); transferred onto PVDF membranes (Millipore); probed using the indicated primary antibodies; and isotype-matched secondary antibodies were conjugated to horseradish peroxidase (Amersham) and detected using ECL (Amersham). The following antibodies were employed: rabbit anti-ERK (1:1,000, Cell Signaling); rabbit anti-pERK (1:1,000, Cell Signaling), mouse anti-COX IV (1:1,000, Molecular Probes); mouse anti-MFN2 (1:1,000, Abnova); mouse anti-ACTIN (1:5,000, Chemicon) chicken anti-MFN1 (1:500, Abcam); rabbit anti-BAK (1:1,000, Millipore); mouse anti-FLAG (1:1,000, Sigma), p-MFN1 (custom made from 21st century biochemical) Densitometric quantification of western blots was performed using the Gel Pro Analyzer 4 or ImageJ softwares.

Immunostaining

For cytochrome *c* immunostaining, 2 × 10^4 MEFs of the indicated genotype grown on 13 mm round coverslips were transfected with as indicated and after 24 hr incubated as detailed in the text, fixed for 30 min at room temperature with 3.7% (V/V) ice-cold formaldehyde, permeabilized for 20 min with 0.01% (V/V) ice-cold Nonidet P-40, and incubated for 15 min with a 0.5% solution of BSA. Cells were then sequentially incubated for 60 min at 37°C with a mouse monoclonal anti-cytochrome *c* antibody (PharMingen, CA, clone 6h2.B4) and for 15 min at 37°C with a FITC-conjugated goat anti-mouse IgG.

For BAX translocation, cells were transfected with mtRFP, fixed in 2% paraformaldehyde for 15 min at room temperature, and then washed five times with PBS. Samples were permeabilized in 0.5% CHAPS for 30 min, followed by incubation in blocking buffer (3% BSA in PBS) for 1 hr at room temperature. Primary antibody (anti-BAX antibody 6A7; 5 μg/ml in blocking buffer) was added and incubated overnight at 4°C, after which Alexa 488-conjugated goat anti-mouse secondary antibody (diluted 1:300 in blocking buffer) was added for 1 hr at room temperature.

Imaging

For confocal microscopy imaging of live cells, 1.8 × 10^5 cells seeded onto 24-mm round glass coverslips transfected and treated as indicated were incubated in Hank's Balanced Salt Solution (HBSS) supplemented with 10 mM HEPES and coverslips were placed on the stage of a Zeiss LSM 510 inverted microscope. Cells expressing mtYFP and mtRFP were excited using the 488 nm or 543 nm line of the Argon laser using a 63c × c1.4 NA Plan Apochromat objective (Zeiss).

For the mitochondrial docking assay (Ishihara et al., 2004), mitochondria from cells of the indicated genotype expressing mtRFP or mtYFP were isolated and resuspended in EB (0.5 mg/ml) at 25°C for 30 min with 1 mM GTP unless indicated. Mitochondria expressing mtRFP or mtYFP were mixed in 1:1 ratio, centrifuged, and incubated for 15 min. Mixed mitochondria were mounted on a 24-mm round coverslip, allowed to attach for 15 min, placed on the stage of a Leica SP5 confocal inverted microscope and excited using the 488 nm or 543 nm line of an Ar laser using a 63c × c1.4 NA Plan Apochromat objective (Leica).

Statistical Analysis

Data are presented as mean ± SEM of the indicated number of independent experiments. p values refer to two tailed Student's t tests between the indicated samples.

SUPPLEMENTAL INFORMATION

Supplemental Information includes five figures and Supplemental Experimental Procedures and can be found with this article online at http://dx.doi.org/10.1016/j.molcel.2015.02.021.

ACKNOWLEDGMENTS

We thank Jean Charron (Centre Hospitalier Universitaire de Québec, Canada) for *Mek1*$^{-/-}$ and *Mek2*$^{-/-}$ cells; Chris Marshall (Institute of Cancer Research, London, UK) for MEK^{DN} and MEK^{CA} plasmids; and Dominique Klein (Friedrich Miescher Institute), Elizabeth Murphy, Marjan Gueck, and Mark Kohr (NHLBI, NIH, USA) for help with mass spectrometry. L.S. is a Senior Scientist of the Dulbecco Telethon Institute. This research was supported by Telethon Italy GGP12162, GPP10005, AIRC Italy, ERC ERMITO, FP7 CIG CristOpa, MIUR FIRB Automed, SNF 31003AB (to L.S.).

Received: July 29, 2014
Revised: January 9, 2015
Accepted: February 12, 2015
Published: March 19, 2015

REFERENCES

Abramov, A.Y., Fraley, C., Diao, C.T., Winkfein, R., Colicos, M.A., Duchen, M.R., French, R.J., and Pavlov, E. (2007). Targeted polyphosphatase expression alters mitochondrial metabolism and inhibits calcium-dependent cell death. Proc. Natl. Acad. Sci. USA *104*, 18091–18096.

Arnoult, D., Grodet, A., Lee, Y.J., Estaquier, J., and Blackstone, C. (2005a). Release of OPA1 during apoptosis participates in the rapid and complete release of cytochrome c and subsequent mitochondrial fragmentation. J. Biol. Chem. *280*, 35742–35750.

Arnoult, D., Rismanchi, N., Grodet, A., Roberts, R.G., Seeburg, D.P., Estaquier, J., Sheng, M., and Blackstone, C. (2005b). Bax/Bak-dependent release of DDP/TIMM8a promotes Drp1-mediated mitochondrial fission and mitoptosis during programmed cell death. Curr. Biol. *15*, 2112–2118.

Beausoleil, S.A., Villén, J., Gerber, S.A., Rush, J., and Gygi, S.P. (2006). A probability-based approach for high-throughput protein phosphorylation analysis and site localization. Nat. Biotechnol. *24*, 1285–1292.

Brooks, C., Wei, Q., Feng, L., Dong, G., Tao, Y., Mei, L., Xie, Z.J., and Dong, Z. (2007). Bak regulates mitochondrial morphology and pathology during apoptosis by interacting with mitofusins. Proc. Natl. Acad. Sci. USA *104*, 11649–11654.

Campello, S., and Scorrano, L. (2010). Mitochondrial shape changes: orchestrating cell pathophysiology. EMBO Rep. *11*, 678–684.

Cereghetti, G.M., Stangherlin, A., Martins de Brito, O., Chang, C.R., Blackstone, C., Bernardi, P., and Scorrano, L. (2008). Dephosphorylation by calcineurin regulates translocation of Drp1 to mitochondria. Proc. Natl. Acad. Sci. USA *105*, 15803–15808.

Cereghetti, G.M., Costa, V., and Scorrano, L. (2010). Inhibition of Drp1-dependent mitochondrial fragmentation and apoptosis by a polypeptide antagonist of calcineurin. Cell Death Differ. *17*, 1785–1794.

Chen, Y., and Dorn, G.W., 2nd. (2013). PINK1-phosphorylated mitofusin 2 is a Parkin receptor for culling damaged mitochondria. Science *340*, 471–475.

Chen, H., Detmer, S.A., Ewald, A.J., Griffin, E.E., Fraser, S.E., and Chan, D.C. (2003). Mitofusins Mfn1 and Mfn2 coordinately regulate mitochondrial fusion and are essential for embryonic development. J. Cell Biol. *160*, 189–200.

Cheng, E.H., Wei, M.C., Weiler, S., Flavell, R.A., Mak, T.W., Lindsten, T., and Korsmeyer, S.J. (2001). BCL-2, BCL-X(L) sequester BH3 domain-only molecules preventing BAX- and BAK-mediated mitochondrial apoptosis. Mol. Cell *8*, 705–711.

Cipolat, S., Martins de Brito, O., Dal Zilio, B., and Scorrano, L. (2004). OPA1 requires mitofusin 1 to promote mitochondrial fusion. Proc. Natl. Acad. Sci. USA *101*, 15927–15932.

Clark-Lewis, I., Sanghera, J.S., and Pelech, S.L. (1991). Definition of a consensus sequence for peptide substrate recognition by p44mpk, the meiosis-activated myelin basic protein kinase. J. Biol. Chem. *266*, 15180–15184.

Cogliati, S., Frezza, C., Soriano, M.E., Varanita, T., Quintana-Cabrera, R., Corrado, M., Cipolat, S., Costa, V., Casarin, A., Gomes, L.C., et al. (2013). Mitochondrial cristae shape determines respiratory chain supercomplexes assembly and respiratory efficiency. Cell *155*, 160–171.

Cribbs, J.T., and Strack, S. (2007). Reversible phosphorylation of Drp1 by cyclic AMP-dependent protein kinase and calcineurin regulates mitochondrial fission and cell death. EMBO Rep. *8*, 939–944.

Danial, N.N., and Korsmeyer, S.J. (2004). Cell death: critical control points. Cell *116*, 205–219.

de Brito, O.M., and Scorrano, L. (2008). Mitofusin 2 tethers endoplasmic reticulum to mitochondria. Nature *456*, 605–610.

DeVay, R.M., Dominguez-Ramirez, L., Lackner, L.L., Hoppins, S., Stahlberg, H., and Nunnari, J. (2009). Coassembly of Mgm1 isoforms requires cardiolipin and mediates mitochondrial inner membrane fusion. J. Cell Biol. *186*, 793–803.

Dhillon, A.S., Hagan, S., Rath, O., and Kolch, W. (2007). MAP kinase signalling pathways in cancer. Oncogene *26*, 3279–3290.

Duvezin-Caubet, S., Jagasia, R., Wagener, J., Hofmann, S., Trifunovic, A., Hansson, A., Chomyn, A., Bauer, M.F., Attardi, G., Larsson, N.G., et al. (2006). Proteolytic processing of OPA1 links mitochondrial dysfunction to alterations in mitochondrial morphology. J. Biol. Chem. *281*, 37972–37979.

Frank, S., Gaume, B., Bergmann-Leitner, E.S., Leitner, W.W., Robert, E.G., Catez, F., Smith, C.L., and Youle, R.J. (2001). The role of dynamin-related protein 1, a mediator of mitochondrial fission, in apoptosis. Dev. Cell *1*, 515–525.

Frezza, C., Cipolat, S., Martins de Brito, O., Micaroni, M., Beznoussenko, G.V., Rudka, T., Bartoli, D., Polishuck, R.S., Danial, N.N., De Strooper, B., and Scorrano, L. (2006). OPA1 controls apoptotic cristae remodeling independently from mitochondrial fusion. Cell *126*, 177–189.

Frezza, C., Cipolat, S., and Scorrano, L. (2007). Organelle isolation: functional mitochondria from mouse liver, muscle and cultured fibroblasts. Nat. Protoc. *2*, 287–295.

Gao, D.K., Huang, T., Jiang, X.F., Hu, S.J., Zhang, L., and Fei, Z. (2014). Resveratrol protects primary cortical neuron cultures from transient oxygen-glucose deprivation by inhibiting MMP-9. Mol Med Rep *9*, 2197–2204.

Germain, M., Mathai, J.P., McBride, H.M., and Shore, G.C. (2005). Endoplasmic reticulum BIK initiates DRP1-regulated remodelling of mitochondrial cristae during apoptosis. EMBO J. *24*, 1546–1556.

Glauser, L., Sonnay, S., Stafa, K., and Moore, D.J. (2011). Parkin promotes the ubiquitination and degradation of the mitochondrial fusion factor mitofusin 1. J. Neurochem. *118*, 636–645.

Gomes, L.C., Di Benedetto, G., and Scorrano, L. (2011). During autophagy mitochondria elongate, are spared from degradation and sustain cell viability. Nat. Cell Biol. *13*, 589–598.

Green, D.R., and Kroemer, G. (2004). The pathophysiology of mitochondrial cell death. Science *305*, 626–629.

Ishihara, N., Eura, Y., and Mihara, K. (2004). Mitofusin 1 and 2 play distinct roles in mitochondrial fusion reactions via GTPase activity. J. Cell Sci. *117*, 6535–6546.

Karbowski, M., Arnoult, D., Chen, H., Chan, D.C., Smith, C.L., and Youle, R.J. (2004). Quantitation of mitochondrial dynamics by photolabeling of individual organelles shows that mitochondrial fusion is blocked during the Bax activation phase of apoptosis. J. Cell Biol. *164*, 493–499.

Karbowski, M., Norris, K.L., Cleland, M.M., Jeong, S.Y., and Youle, R.J. (2006). Role of Bax and Bak in mitochondrial morphogenesis. Nature *443*, 658–662.

Kodiha, M., Bański, P., and Stochaj, U. (2009). Interplay between MEK and PI3 kinase signaling regulates the subcellular localization of protein kinases ERK1/2 and Akt upon oxidative stress. FEBS Lett. *583*, 1987–1993.

Koshiba, T., Detmer, S.A., Kaiser, J.T., Chen, H., McCaffery, J.M., and Chan, D.C. (2004). Structural basis of mitochondrial tethering by mitofusin complexes. Science *305*, 858–862.

Leboucher, G.P., Tsai, Y.C., Yang, M., Shaw, K.C., Zhou, M., Veenstra, T.D., Glickman, M.H., and Weissman, A.M. (2012). Stress-induced phosphorylation and proteasomal degradation of mitofusin 2 facilitates mitochondrial fragmentation and apoptosis. Mol. Cell *47*, 547–557.

McCubrey, J.A., Steelman, L.S., Chappell, W.H., Abrams, S.L., Wong, E.W.T., Chang, F., Lehmann, B., Terrian, D.M., Milella, M., Tafuri, A., et al. (2007). Roles of the Raf/MEK/ERK pathway in cell growth, malignant transformation and drug resistance. Biochim. Biophys. Acta *1773*, 1263–1284.

Meeusen, S., Tieu, Q., Wong, E., Weiss, E., Schieltz, D., Yates, J.R., and Nunnari, J. (1999). Mgm101p is a novel component of the mitochondrial nucleoid that binds DNA and is required for the repair of oxidatively damaged mitochondrial DNA. J. Cell Biol. *145*, 291–304.

Montessuit, S., Somasekharan, S.P., Terrones, O., Lucken-Ardjomande, S., Herzig, S., Schwarzenbacher, R., Manstein, D.J., Bossy-Wetzel, E., Basañez, G., Meda, P., and Martinou, J.C. (2010). Membrane remodeling induced by the dynamin-related protein Drp1 stimulates Bax oligomerization. Cell *142*, 889–901.

Otera, H., Wang, C., Cleland, M.M., Setoguchi, K., Yokota, S., Youle, R.J., and Mihara, K. (2010). Mff is an essential factor for mitochondrial recruitment of Drp1 during mitochondrial fission in mammalian cells. J. Cell Biol. *191*, 1141–1158.

Otera, H., Ishihara, N., and Mihara, K. (2013). New insights into the function and regulation of mitochondrial fission. Biochim. Biophys. Acta *1833*, 1256–1268.

Pagès, G., Brunet, A., L'Allemain, G., and Pouysségur, J. (1994). Constitutive mutant and putative regulatory serine phosphorylation site of mammalian MAP kinase kinase (MEK1). EMBO J. *13*, 3003–3010.

Rambold, A.S., Kostelecky, B., Elia, N., and Lippincott-Schwartz, J. (2011). Tubular network formation protects mitochondria from autophagosomal degradation during nutrient starvation. Proc. Natl. Acad. Sci. USA *108*, 10190–10195.

Rasola, A., Sciacovelli, M., Chiara, F., Pantic, B., Brusilow, W.S., and Bernardi, P. (2010). Activation of mitochondrial ERK protects cancer cells from death through inhibition of the permeability transition. Proc. Natl. Acad. Sci. USA *107*, 726–731.

Scorrano, L., Ashiya, M., Buttle, K., Weiler, S., Oakes, S.A., Mannella, C.A., and Korsmeyer, S.J. (2002). A distinct pathway remodels mitochondrial cristae and mobilizes cytochrome c during apoptosis. Dev. Cell *2*, 55–67.

Scorrano, L., Oakes, S.A., Opferman, J.T., Cheng, E.H., Sorcinelli, M.D., Pozzan, T., and Korsmeyer, S.J. (2003). BAX and BAK regulation of endoplasmic reticulum Ca2+: a control point for apoptosis. Science *300*, 135–139.

Shaul, Y.D., and Seger, R. (2007). The MEK/ERK cascade: from signaling specificity to diverse functions. Biochim. Biophys. Acta *1773*, 1213–1226.

Stanciu, M., Wang, Y., Kentor, R., Burke, N., Watkins, S., Kress, G., Reynolds, I., Klann, E., Angiolieri, M.R., Johnson, J.W., and DeFranco, D.B. (2000). Persistent activation of ERK contributes to glutamate-induced oxidative toxicity in a neuronal cell line and primary cortical neuron cultures. J. Biol. Chem. *275*, 12200–12206.

Tondera, D., Grandemange, S., Jourdain, A., Karbowski, M., Mattenberger, Y., Herzig, S., Da Cruz, S., Clerc, P., Raschke, I., Merkwirth, C., et al. (2009). SLP-2 is required for stress-induced mitochondrial hyperfusion. EMBO J. *28*, 1589–1600.

Wang, Z.G., Jiang, H., Chen, S., Du, F.H., and Wang, X.D. (2012). The mitochondrial phosphatase PGAM5 functions at the convergence point of multiple necrotic death pathways. Cell *148*, 228–243.

Wei, M.C., Zong, W.X., Cheng, E.H., Lindsten, T., Panoutsakopoulou, V., Ross, A.J., Roth, K.A., MacGregor, G.R., Thompson, C.B., and Korsmeyer, S.J. (2001). Proapoptotic BAX and BAK: a requisite gateway to mitochondrial dysfunction and death. Science *292*, 727–730.

Westermann, B. (2010). Mitochondrial fusion and fission in cell life and death. Nat. Rev. Mol. Cell Biol. *11*, 872–884.

Yung, H.W., and Tolkovsky, A.M. (2003). Erasure of kinase phosphorylation in astrocytes during oxygen-glucose deprivation is controlled by ATP levels and activation of phosphatases. J. Neurochem. *86*, 1281–1288.

Ziviani, E., Tao, R.N., and Whitworth, A.J. (2010). Drosophila parkin requires PINK1 for mitochondrial translocation and ubiquitinates mitofusin. Proc. Natl. Acad. Sci. USA *107*, 5018–5023.

Molecular Cell, Volume *58*

Supplemental Information

Extracellular Regulated Kinase Phosphorylates Mitofusin 1 to Control Mitochondrial Morphology and Apoptosis

Aswin Pyakurel, Claudia Savoia, Daniel Hess, and Luca Scorrano

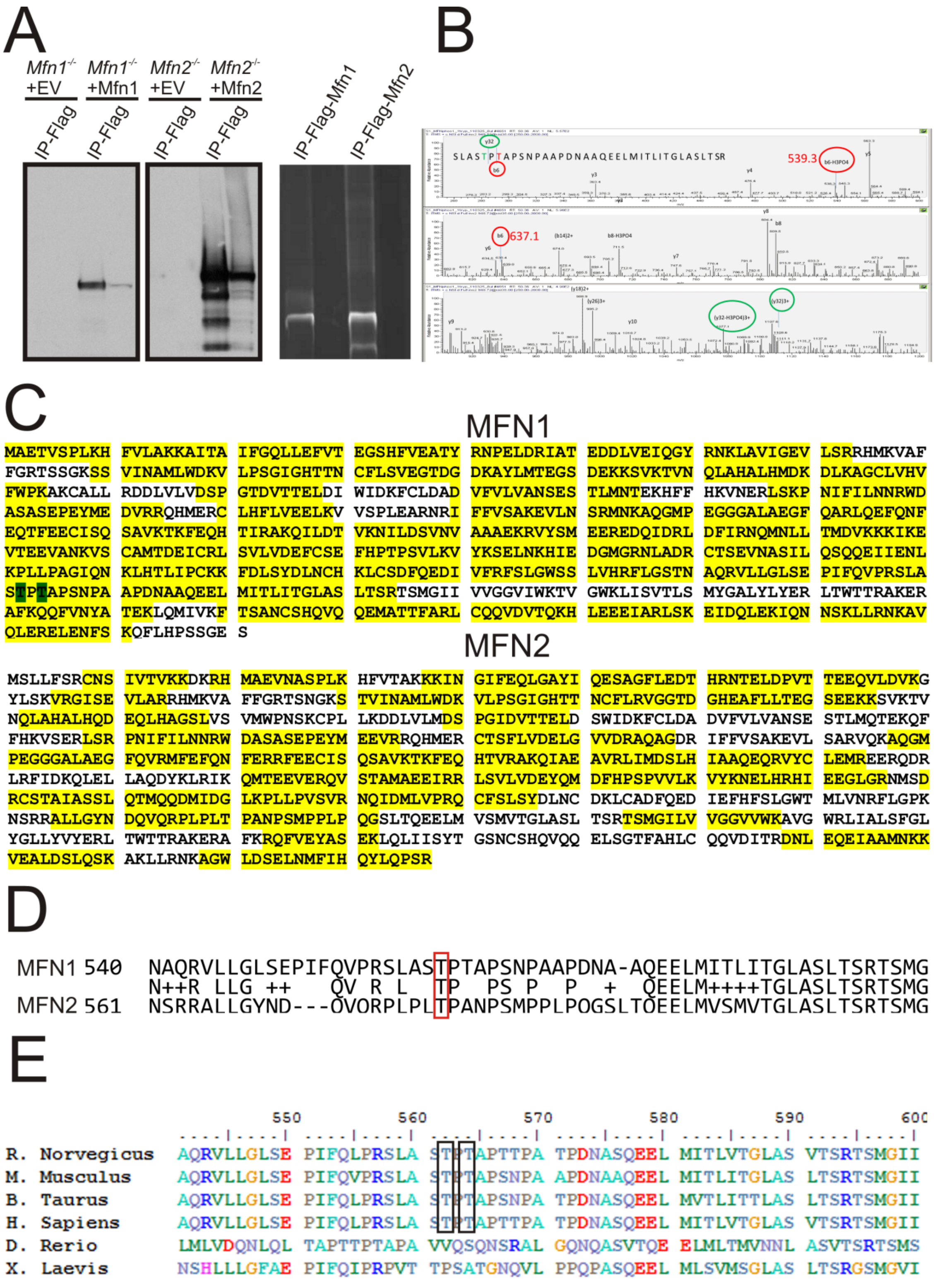

Figure S1. LC-MS/MS Identification of MFN1 Phosphorylation, Related to Fig. 1

(A) FLAG-MFN1 and FLAG-MFN2 were immunoprecipitated where indicated from *Mfn1*$^{-/-}$ and *Mfn2*$^{-/-}$ MEFs transfected as indicated, separated by SDS PAGE and detected by anti-Flag immunoblotting (left panels) or Simply Blue Safe staining (right panel).

(B) MS/MS spectrum of the tryptic peptide of MFN1 containing one phosphorylation either on T562 or T564. Ions highlighted with green circles account for the T562 phosphorylation whereas the ion highlighted with a red circle accounts for the T564 phosphorylation.

(C) LC-MS/MS coverage of the immune-purified FLAG-MFN1 and FLAG-MFN2. Detected peptides are highlighted in yellow, post-translational modifications in green.

(D) ClustalW alignment of the phosphorylated region of MFN1 with MFN2. Box: conserved T

(E) ClustalW alignment of the phosphorylated region of MFN1 of the indicated species. Box: conserved phosphorylated T

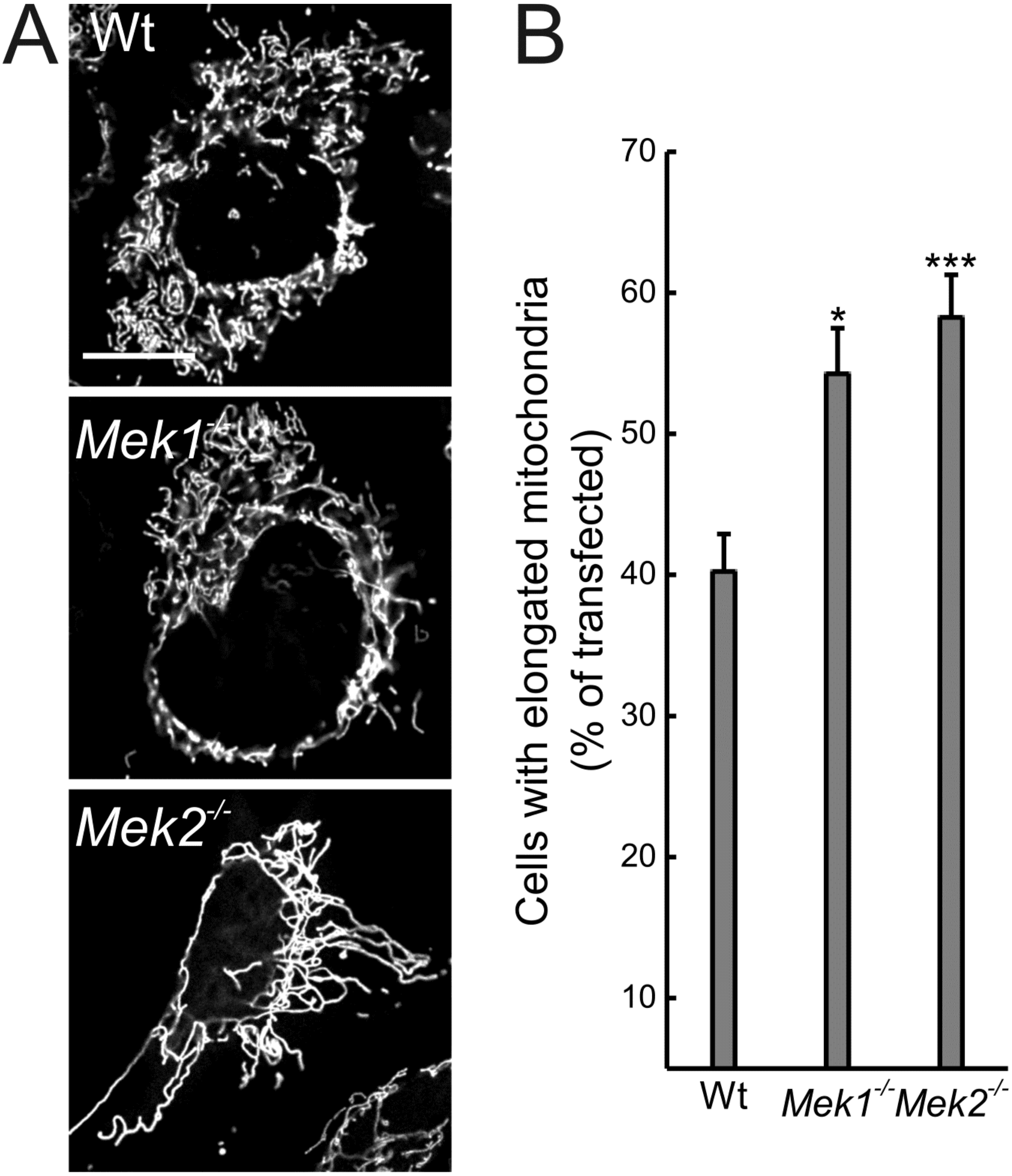

Figure S2. Mitochondria Are Elongated In *Mek1*$^{-/-}$ and *Mek2*$^{-/-}$ MEFs, Related to Fig. 2

(A) Representative confocal images of mitochondrial morphology in MEFs of the indicated genotype transfected with mtYFP. Scale Bar, 10 µm.

(B) Experiments were carried out as in **A**. Data represent mean±SEM of 5 independent experiments (n=100 cells per condition in each experiment). *P<0.05, **P<0.01, ***P<0.001 in a paired Student's t Test vs. Wt

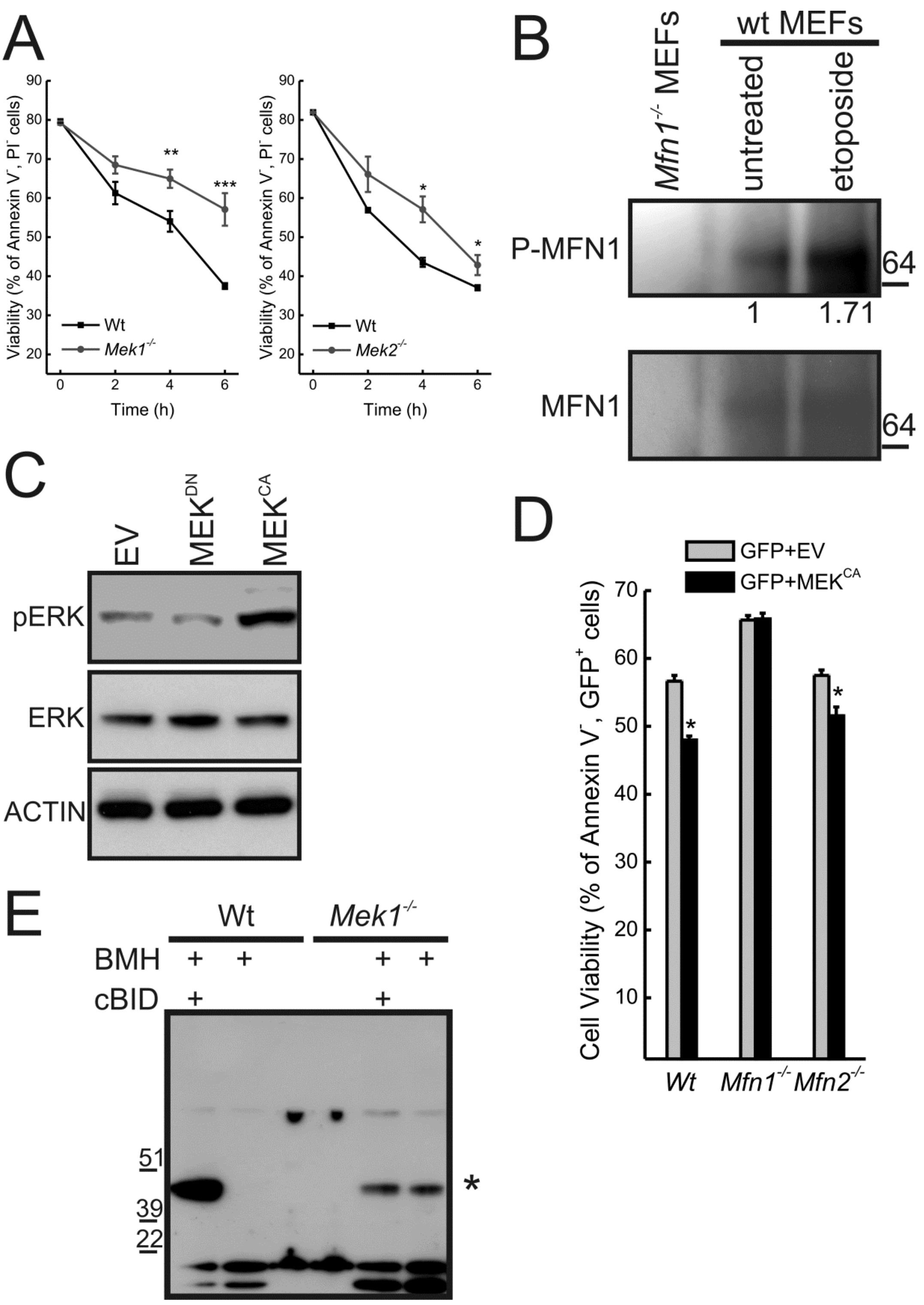

Figure S3. MFN1 Phosphorylation Occurs during and Modulates Cell Death, Related to Fig. 3

(A) MEFs of indicated genotypes were treated with 2 μM staurosporine for the indicated times. Data are mean±SEM of 5 independent experiments. *P<0.05, **P<0.01, ***P<0.001 in a paired Student's t Test vs. Wt

(B) Endogenous MFN1 was immunoprecipitated from MEFs of the indicated genotype treated where indicated with 2 μM etoposide, separated by SDS-PAGE and immunoblotted using the indicated antibodies. The densitometric P-MFN1/MFN1 ratio is indicated, normalized to untreated MEFs.

(C) MEFs were transfected with the indicated plasmids and lysed after 24 hrs. Equal amounts (30 μg) of proteins were separated by SDS-PAGE and immunoblotted using the indicated antibodies.

(D) MEFs of indicated genotypes cotransfected with GFP and the indicated plasmids were treated with 2 μM staurosporine for 6 hours. Data represent mean±SEM of 3 independent experiments. *P<0.05, **P<0.01, ***P<0.001 in a paired Student's t Test vs. Wt

(E) Mitochondria isolated from the indicated genotypes were treated where indicated with cBID and crosslinked with 10mM BMH where indicated. Equal amounts (30 μg) of proteins were analyzed by SDS-PAGE/immunoblotting. Asterisk: BAK multimers.

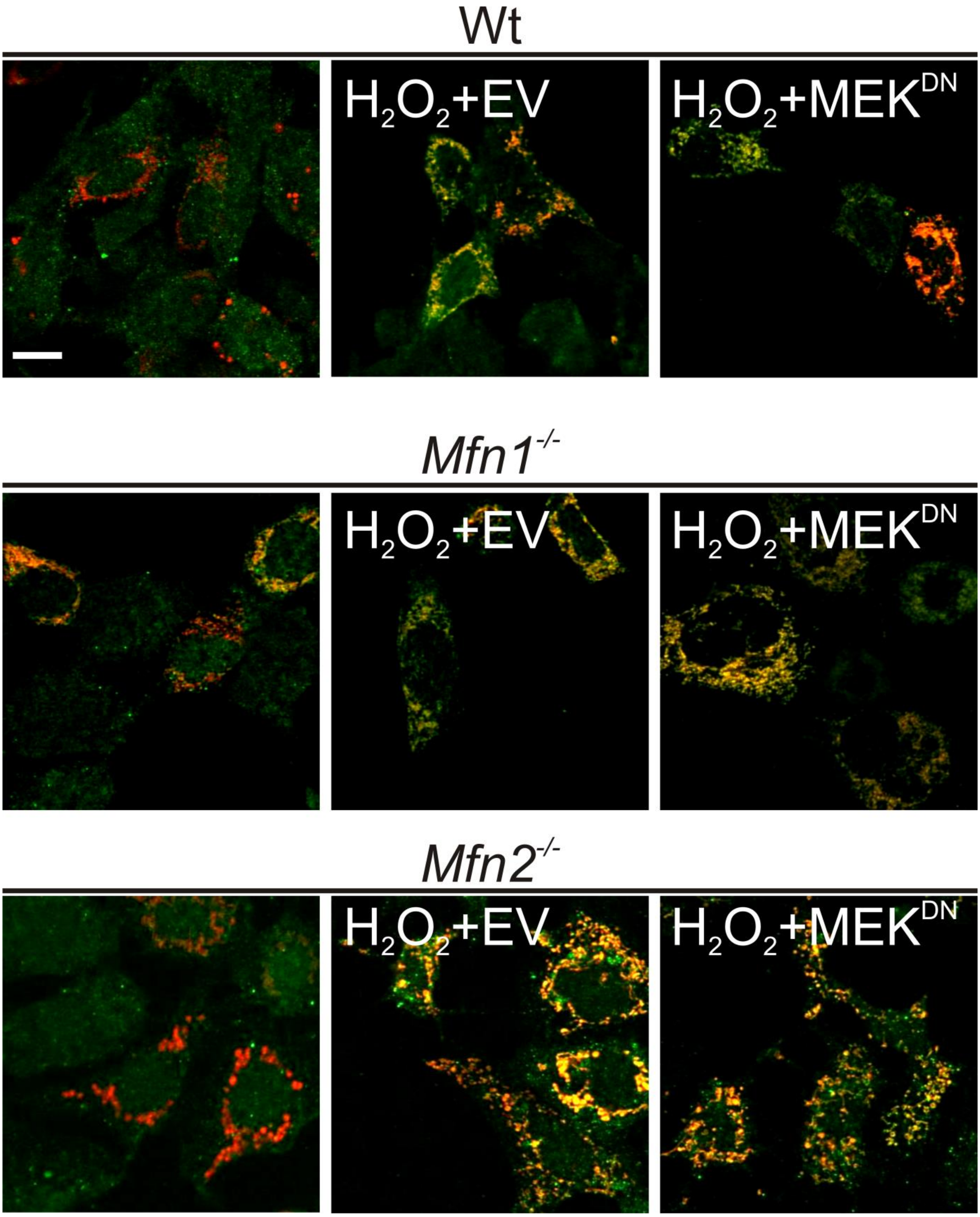

Figure S4. MEK^{DN} Does Not Alter BAX Translocation, Related to Fig. 4.
Representative images of subcellular BAX distribution in MEFs of indicated genotypes cotransfected with mtRFP (red) and the indicated plasmids and treated where indicated for 30 min with 1 mM H_2O_2, fixed and immunostained for BAX (green). Scale Bar, 10 µm.

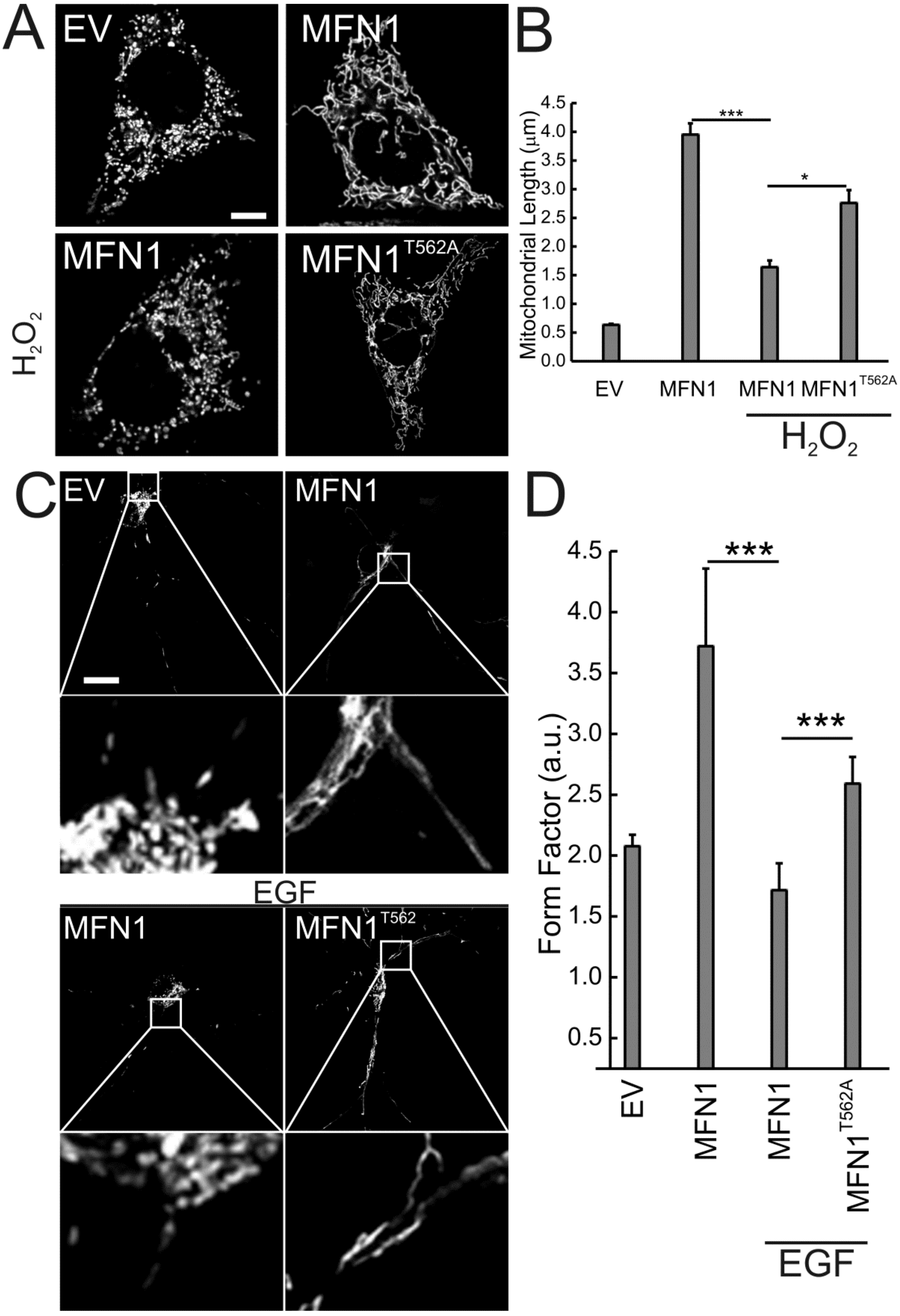

Figure S5. MFN1 Phosphorylation Regulates Mitochondrial Morphology in Oxidative Stress and Primary Cortical Neurons, Related to Fig. 5

(A) Representative confocal images of mitochondrial morphology in MEFs of the indicated genotype cotransfected with mtYFP and the indicated plasmids. Cells were treated with H_2O_2 (250μM for 30 min) where indicated. Scale bar, 10 μm.

(B) Experiments were carried out as in (A). Data represent mean±SEM of 4 independent experiments. *P<0.05, ***P<0.001 in a paired Student's t Test between the indicated treatments

(C) Representative images of primary cortical neurons co-transfected with mtRFP and the indicated plasmids. Where indicated, neurons were treated with 10 nM EGF for 10 min. Scale bar, 5 μm. The boxed areas are magnified 6X.

(D) Quantitative analysis of mitochondrial morphology in experiments carried out as in (A). Data represent mean±SEM of 4 independent experiments. ***P<0.001 in a paired Student's t Test between the indicated treatments.

Supplemental Experimental Procedures

Molecular Biology

For the generation of the Mfn1 mutants, the following primers were used:

T562A F: 5'–GTCCCTAGATCTTTAGCTTCAGCTCCTACTGCTCCTTCTAACC-3'; R: 5'-GGTTAGAAGGAGCAGTAGGAGCTGAAGCTAAAGATCTAGGGAC-3,

T562D F: 5'-CCCTAGATCTTTAGCTTCAGACCCTACTGCTCCTTCTAACC-3'; R: 5'-GGTTAGAAGGAGCAGTAGGGTCTGAAGCTAAAGATCTAGGG-3',

T564A F: 5'-ATCTTTAGCTTCAACTCCTACTGCTCCTTCTAACCCAGC-3'; R: 5'-GCTGGGTTAGAAGGAGCAGTAGGAGTTGAAGCTAAAGAT-3'),

T564D F: 5'-TCTTTAGCTTCAGACCCTGATGCTCCTTCTAACCCAGC-3'; R: 5'-GCTGGGTTAGAAGGAGCATCAGGGTCTGAAGCTAAAGA-3',

T562AT564A F: 5'-GTCCCTAGATCTTTAGCTTCAGCTCCTGCTGCTCCTTCTAACC-3'; R: 5'-GGTTAGAAGGAGCAGCAGGAGCTGAAGCTAAAGATCTAGGGAC-3',

T562DT564D F: 5'-CCCTAGATCTTTAGCTTCAGACCCTGATGCTCCTTCTAACC-3'; R: 5'-GGTTAGAAGGAGCATCAGGGTCTGAAGCTAAAGATCTAGGG-3'.

Liquid chromatography-mass spectrometry

Extracted peptides from gels were injected onto a reversed phase column for liquid chromatography-mass spectrometry (LC-MS) analysis in the information-dependent acquisition mode. Electrospray ionization LC-MS/MS was performed using a Magic C18 HPLC column (75 µm × 10 cm; Swiss BioAnalytics) with a 1200 Nano-HPLC system (Agilent Technologies) connected to a LTQ Orbitrap Velos (Thermo Scientific). The peptides were loaded onto a peptide captrap (Michrom BioResources) at a flow rate of 10 µl / min for 5 minutes. They were eluted at a flow rate of 400 nl / min with a linear gradient of 2 – 36 %

acetonitrile in 0.1 % formic acid (in H_2O) in 30 minutes. Information-dependent acquisition analyses was done according to the manufacturer's recommendations, i.e. 1 survey scan at 60K resolution in the Orbitrap cell was followed by up to 20 product ion scans in the linear ion trap, and precursors were excluded for 15s after their second occurrence. Individual MS/MS spectra, containing sequence information were compared with the program Mascot against the mammalian sub set of the protein sequence database Swiss-Prot 2010_09 (Perkins *et al.*, 1999). Carboxyamidomethylation of cysteine (+57.0245 Da) was set as a fixed modification and phosphorylation of serine, threonine and tyrosine (+79.9663 Da), oxidation of methionine (+15.9949 Da), deamidation of asparagine and glutamine (+0.984016 Da) and Pyro-glu formation on N-terminal glutamine (-17.02655 Da) were set as variable. Parent tolerance was 3 PPM and fragment tolerance 0.8 Da. No enzyme specificity was used for the combined searches of the tryptic and AspN digested samples. The results were further analyzed with Scaffold and ScaffoldPTM (Proteome Software).

Combined oxygen and glucose deprivation (OGD) in primary neurons

Primary cortical neurons were exposed to OGD as described (Goldberg and Choi, 1993). Briefly, the culture medium was replaced with a hypoxic medium previously saturated for 20 min with 95% N_2 and 5% CO_2 and containing 116mM NaCl, 5.4mM KCl, 0.8mM $MgSO_4$, 26.2mM $NaHCO_3$, 1mM NaH_2PO_4 , 1.8mM $CaCl_2$, 0.01mM glycine and 0.001w/v phenol red . Hypoxic conditions were maintained using a hypoxia chamber (1 hour, 37°C, 95% N_2/5% CO_2). These experimental conditions induced a 30% pO_2 decrease in the medium.

Mitochondrial morphology analysis in primary neurons

To label mitochondria, neuronal cells were transfected with mitochondrially-targeted dsRED protein (mtRFP). Digital images were captured on a confocal microscope using a 63X oil immersion lens and, to compensate for optical imperfections of the microscope and to enhance the quality of images, subjected to a 2D deconvolution, using the ‘’Integrative Deconvolution’’ plug-in for ImageJ software. Then, images were analyzed using the ImageJ macro "Morphometry" described in (Cribbs and Strack, 2009) to determine the two mitochondrial shape metrics form factor (FF) and aspect ratio (AR).

Cell death in primary cortical neurons

For TUNEL assays, primary neurons transfected and treated as indicated were seeded onto 13mm round glass coverslips, fixed, immunostained with anti-β-Tubulin III antibody (1:400, Sigma) and an isotype-matched Alexa-647 secondary antibody, and incubated in TUNEL reagent (Roche) following manufacturer’s instructions. Coverslips were mounted on slides with a DAPI-containing AntiFade ProGold mounting solution (Invitrogen). For DAPI, TUNEL, mtRFP and β-Tubulin III detection, coverslips were excited using the Diode 405/30 nm, Argon/2 488/514nm and HeNe 633nm lasers, respectively. Images were acquired sequentially using four separate color channels using of a Zeiss LSM Meta using a 63x Oil DIC EC Plan Neofluar objective (Zeiss). Cell death was measured as the percentage of TUNEL positive cells in the mtRPF/β-Tubulin III positive population. A total of 30 neurons were counted per condition in each independent experiment.

Supplemental References

Cribbs,J.T. and Strack,S. (2009). Functional characterization of phosphorylation sites in dynamin-related protein 1. Methods Enzymol. *457*, 231-253.

Goldberg,M.P. and Choi,D.W. (1993). Combined oxygen and glucose deprivation in cortical cell culture: calcium-dependent and calcium-independent mechanisms of neuronal injury. J. Neurosci. *13*, 3510-3524.

Perkins,D.N., Pappin,D.J.C., Creasy,D.M., and Cottrell,J.S. (1999). Probability-based protein identification by searching sequence databases using mass spectrometry data. Electrophoresis *20*, 3551-3567.
